# In silico activity and ADMET profiling of phytochemicals from Ethiopian indigenous aloes using pharmacophore models

**DOI:** 10.1038/s41598-022-26446-x

**Published:** 2022-12-23

**Authors:** Lemessa Etana Bultum, Gemechu Bekele Tolossa, Gwangmin Kim, Ohhyeon Kwon, Doheon Lee

**Affiliations:** 1 Department of Bio and Brain Engineering, Korea Advanced Institute of Science and Technology (KAIST), 291Daehak-Ro, Daejeon, 34141 South Korea; 2 Bio-Synergy Research Center, 291Daehak-Ro, Daejeon, 34141 South Korea; 3 Department of Applied Bioscience, Dong-A Universtiy, Busan 49315, South Korea; 4 Department of Neuroscience, Washington University School of Medicine, St. Louis, MO 63110 USA

**Keywords:** Biochemistry, Computational biology and bioinformatics, Drug discovery, Systems biology, Medical research

## Abstract

In silico profiling is used in identification of active compounds and guide rational use of traditional medicines. Previous studies on Ethiopian indigenous aloes focused on documentation of phytochemical compositions and traditional uses. In this study, ADMET and drug-likeness properties of phytochemicals from Ethiopian indigenous aloes were evaluated, and pharmacophore-based profiling was done using Discovery Studio to predict therapeutic targets. The targets were examined using KEGG pathway, gene ontology and network analysis. Using random-walk with restart algorithm, network propagation was performed in CODA network to find diseases associated with the targets. As a result, 82 human targets were predicted and found to be involved in several molecular functions and biological processes. The targets also were linked to various cancers and diseases of immune system, metabolism, neurological system, musculoskeletal system, digestive system, hematologic, infectious, mouth and dental, and congenital disorder of metabolism. 207 KEGG pathways were enriched with the targets, and the main pathways were metabolism of steroid hormone biosynthesis, lipid and atherosclerosis, chemical carcinogenesis, and pathways in cancer. In conclusion, in silico target fishing and network analysis revealed therapeutic activities of the phytochemicals, demonstrating that Ethiopian indigenous aloes exhibit polypharmacology effects on numerous genes and signaling pathways linked to many diseases.

## Introduction

Healthcare systems around the world are encountering increased levels of pandemics, chronic illnesses, population aging and escalating healthcare costs. Patients and healthcare providers alike are demanding that healthcare services be revitalized. This includes improving access to traditional, complementary, and alternative medicine (TCAM) practitioners, products, and services, particularly in primary healthcare^1^. Natural products’ bioactive components have been widely used for treating a broad spectrum of diseases for centuries and are considered as promising alternative therapeutic agents due to their potential healing effects^2^. Over the past three decades, there has been a significant increase in the usage of natural product medicines, with 80% of people globally relying on them for some aspect of basic healthcare^3^. The use of plants as a source of natural products and as the basis for traditional medicine systems across many different cultures has been extensively documented for thousands of years^2,4,5^.

Plants of the genus Aloe are the most common traditional medicinal plants in recorded history ^6^. According to literature records, 74% of the uses of the genus Aloe were as medicine, and exudate was frequently used in these applications^7^. Aloe leaves have been used for thousands of years as a primary remedy for skin conditions, burns, and constipation^8^. Additionally, there are numerous commercial products that contain aloe species, such as laxative medications, health beverages and tonics, after-shave gel, mouthwash and toothpaste, hair tonic and shampoo, and skin-moisturizing gels^9,10^. Aloes were important in Ethiopia for their therapeutic properties as well as for securing livelihoods, fostering economic growth, and protecting biodiversity^9^. With 46 known aloe species and three subspecies, Ethiopia is one of the likely centers of aloe biodiversity. Of these species, 67.3% are indigenous to the nation^11,12^. In Ethiopia, three regional centers of endemism have been identified^10^: (1) North and central highlands and west of the great rift valley have endemic species and infraspecific taxa of *Aloe adigratana*, *A. ankoberensis*, *A. benishangulana*, *A. camperi*, *A. clarkei*, *A. debrana*, *A. elegans*, *A. monticola*, *A. percrassa*, A. *pulcherrima*, *A. schelpei*, *A. sinana*, *A. steudneri*, *A. trigonantha*, and *A. weloensis*; (2) Eastern highlands and lowlands contains endemic species of *Aloe bertemariae*, *A. harlana*, *A. mcloughlinii*, *A. megalacantha* subsp. alticola, *A. pirottae*; *A. pubescens*; and *A. trichosantha* subsp. Longiflora; and (3) Southern highlands, lowlands and rift valley with species *Aloe elkerriana*, *A. friisii*, *A. ghibensis*, *A. gilbertii* subsp. gilbertii, *A. gilbertii* subsp. megalacanthoides, *A. jacksonii*, *A. kefaensis*, *A. otallensis*, *A. tewoldei*, *A. welmelensis*, and *A. yavellana*.

A great deal of effort is being done to identify the aloes’ phytochemical compositions because of their extensive medicinal uses for long period of time. Aloe species' leaves and roots are reservoirs for a variety of intriguing secondary metabolites, including alkaloids, anthraquinones, pre-anthraquinones, anthrones, bianthraquinoids, chromones, flavonoids, coumarins, and pyrones^8^. However, major active ingredients, associated diseases, and their mode of action, particularly for indigenous Ethiopian aloes, are not well understood. This hampered their widespread usage in the development of drugs.

Finding bioactive compounds to aid in the treatment of diseases is the goal of the drug development process. In the past, the new drug discovery process begins with random screening and empirical observations of the effects of natural products for known diseases. Currently, this process is improved by high throughput screening (HTS), which enabled the screening process of many thousands of compounds against a molecular target or cellular assay very quickly^13^. Furthermore, researchers are constantly investigating new methods to increase the efficiency of the drug discovery process^14^. Utilizing computer-aided drug design (CADD), often known as the in silico method, is one strategy to improve the effectiveness of novel drug development. Through the use of molecular modeling that enables virtually screening a large number of compounds for drug-likeness properties and their interaction with pharmacological targets, CADD reduces of the drug development time and overall research expenses^13^. Compound in silico activity profiling is an emerging computational method for predicting the most likely targets of a bioactive compound and therefore anticipating adverse reactions, side effects, and drug repurposing^15^. It can be applied in many different fields including drug repositioning, natural product profiling and multitarget approaches^16^. Studies on the new uses of already established drugs are being carried out in addition to classical drug development. In silico profiling of old drugs can help to reposition such old drugs for new uses^17,18^. Compounds that emerge from phenotypic-based screening including empirical knowledge have usually a completely unknown mechanism of action (MoA). Pharmacophore-based activity profiling of natural products enables to predict their mechanism of action and guides in proper planning of in vivo and in vitro experiments. In addition, previously unknown lead therapeutic natural products with interesting chemical scaffolds can be profiled^16^. This will help to prioritize compounds based on their predicted target profile. The understanding and the prediction of the synergistic role of polypharmacology is also one of the main applications of in silico target profiling^19^.

Pharmacophore model-based in silico profiling for the compounds from the Ethiopian indigenous aloes was done in this study. A pharmacophore is an abstract description of the molecular features (both steric and electronic) required for the molecular recognition of ligands by biological macromolecules, influencing their biological processes^20^. Structurally distinct molecules with similar pharmacophore patterns are likely to be recognized by the same binding site of a biological target and thus have similar biological profiles^21^. Pharmacophore models are typically used when several drugs (active compounds) have been identified, but the three-dimensional (3D) structure of the target protein or receptor is unknown. Drugs are superimposed to determine their common properties, providing a pharmacophore model that describes ligand-receptor binding^22^. Any atom or group in a molecule with specific properties related to molecular recognition can be reduced to pharmacophore properties^23^. These molecular patterns could be hydrogen bond donors or acceptors, cationic, anionic, aromatic or hydrophobic, and any possible combination^24^. For a set of active molecules, pharmacophore identification involves two steps: analyzing the molecule to identify its pharmacophore properties, that is, atoms that can interact with a receptor, and aligning the active conformations of the molecules to find the best overlay of the corresponding features^22^.

In this study, ADMET profiling and pharmacophore model-based in silico target prediction of the phytochemicals from Ethiopian indigenous aloes was performed. To identify related diseases and reveal the mechanism underlying the therapeutic effects of aloes' phytochemicals, gene ontology (GO), KEGG pathway^25^, and network analysis of the predicted targets were investigated. To the best of our knowledge, no computerized comprehensive target fishing approach has been used to identify the pharmacological potential of Ethiopian indigenous aloes’ phytochemicals.

## Results and discussion

### Evaluation of physicochemical, drug-likeness and ADMET related properties of phytochemicals from Ethiopian indigenous aloes

Physicochemical properties affect pharmacodynamics and pharmacokinetics of molecules. Absorption, Distribution, Metabolism, Excretion, and Toxicity (ADMET) properties of a molecule are important factors in successful drug and other industrial chemicals development^26,27^. Utilizing physicochemical properties that were compiled from the PubChem database^28^ and SwissADME webserver^29^, the phytochemicals' drug-likeness was assessed. ADMET-related properties of phytochemicals from Ethiopian indigenous aloes were predicted using admetSAR web server—a database which contains ADMET-associated properties curated form a large number of diverse literatures^27^. Prior to in silico profiling of the phytochemicals, we excluded potentially harmful substances that are AMES mutagens, carcinogens, and hERG inhibitors using the data from admetSAR.

We used Lipinski’s rule of 5^30^ and Veber’s rule^31^ to assess the drug-likeness properties of phytochemicals from Ethiopian indigenous aloes. However, it should be noted that many of the most successful drugs do not fit these guidelines^32^. For oral drugs from natural products or natural product derivatives, 2 or 3 rule of five violations are common^33^. As shown in Table 1, the majority of phytochemicals comply with Veber's rule criteria and the rule of five (with a maximum of 3 rule violations). Thus, the phytochemicals found in indigenous Ethiopian aloes demonstrated adequate drug-likeness and physicochemical characteristics indicating their potential as therapeutic medication.

**Table 1:** Lipinski’s rule of five and Veber’s rule for drug-likeness analysis of Ethiopian indigenous aloes’ phytochemicals.

No	Compound	Molecular weight (g/mol)	Lipophilicity (log *p*)	Hydrogen bond donors	Hydrogen bond acceptors	TPSA (Å^2^)	ROTB	Number of Lipinsk’s rules violations	Number of Veber’s rules violations
1	Aloe-emodin	270.24	1.8	3	5	94.8	1	0	0
2	Homonataloin	432.4	1.1	6	9	157	3	0	1
3	Microdontin A	564.5	1.6	7	11	194	7	3	1
4	Microdontin B	550.6	1.6	7	10	177	7	2	1
5	Benzyl salicylate	228.24	3.2	1	3	46.5	4	0	0
6	Diisobutyl phthalate	278.34	4.1	0	4	52.6	8	0	0
7	Ethyl salicylate	166.17	3	1	3	46.5	3	0	0
8	Thiophen-2-yl benzoate	204.25	3.3	0	3	54.5	3	0	0
9	1-Hexadecanol	242.44	7.3	1	1	20.2	14	1	1
10	cis-Linoleic acid	266.4	6	1	2	37.3	13	1	1
11	Methyl 9-methyltetradecanoate	256.42	6.6	0	2	26.3	13	1	1
12	Methyl decanoate	186.29	4.7	0	2	26.3	9	0	0
13	Methyl dodecanoate	214.34	5.8	0	2	26.3	11	0	1
14	Methyl linoleate	294.5	6.9	0	2	26.3	15	1	1
15	Methyl palmitate	270.5	7.9	0	2	26.3	15	1	1
16	Methyl tetradecanoate	242.4	6.8	0	2	26.3	13	0	1
17	Palmitic acid	256.42	6.4	1	2	37.3	14	1	1
18	Undecylenic acid	184.27	3.9	1	2	37.3	9	0	0
19	7-O-Methylaloesin	408.4	− 1	4	9	143	5	0	1
20	Aloenin	410.4	0	5	10	155	5	0	1
21	Orcinyl angelate	206.24	2.9	1	3	46.5	3	0	0
22	(E)-2-Isopropyl-5-methylphenyl 2-methylbut-2-enoate	232.32	4.2	0	2	26.3	4	0	0
23	(R)-Ar-Turmerone	216.32	4	0	1	17.1	4	0	0
24	Ar-Turmerone	216.32	4	0	1	17.1	4	0	0
25	Carvacrol	150.22	3.1	1	1	20.2	1	0	0
26	Curlone	218.33	4	0	1	17.1	4	0	0
27	Diosgenin,dehydro	396.6	6.9	0	2	18.5	0	1	0
28	Hinesol	222.37	3.7	1	1	20.2	1	0	0
29	Nerol	154.25	2.9	1	1	20.2	4	0	0
30	Phytol	296.5	8.2	1	1	20.2	13	1	1
31	Turmerone	218.33	3.3	0	1	17.1	4	0	0
32	Heptadecane	240.5	8.8	0	0	0	14	1	1
33	Pentadecane	212.41	7.7	0	0	0	12	1	1
34	beta-Sitosterol	414.7	9.3	1	1	20.2	6	1	0
35	1-Heptadecene	238.5	9.5	0	0	0	14	1	1

*TPSA* topological polar surface area, *ROTB* number of rotatable bonds.

ADME-related properties such as molecular weight (MW), octanol–water partition coefficients (Log *P*), topological polar surface area (TPSA), water solubility, gastrointestinal absorption (GIA), and blood–brain barrier (BBB) permeability were presented in Table 2. Drug candidates exhibit poor absorption when their TPSA is higher than 140 Å^2^, which is benchmarked for marketed drugs^31,34,35^. TPSA has a positive correlation with mass and the molecules with a mass higher than 500 g/mol were observed to have TPSA beyond the range of 0–140^35^. TPSA values and GIA for the majority of the phytochemicals from indigenous Ethiopian aloes were acceptable. The Log *p* value and Abbott bioavailability score of greater than zero indicate that the phytochemicals have a substantial bioavailability and cross the cell membrane efficiently^36^. GIA and BBB permeability are important properties of a drug that is intended for widespread use. There have been numerous prior researches to improve GI permeation of molecules for orally delivered, poorly absorbed medicines^37^. A significant majority of the phytochemicals from indigenous Ethiopian aloes exhibited acceptable GIA and cross the blood–brain barrier.

**Table 2:** Calculated physicochemical properties of Ethiopian indigenous aloes’ phytochemicals.

No	Compound	Molecular formula	Molecular weight (g/mol)	Water solubility Silicos-IT class	GI absorption	BBB permeant	Abbott bioavailability score
1	Aloe-emodin	C_15_H_10_O_5_	270.24	Soluble	High	No	0.55
2	Homonataloin	C_22_H_24_O_9_	432.4	Soluble	High	No	0.55
3	Microdontin A	C_30_H_28_O_11_	564.5	Soluble	Low	No	0.17
4	Microdontin B	C_30_H_30_O_10_	550.6	Moderately soluble	Low	No	0.17
5	Benzyl salicylate	C_14_H_12_O_3_	228.24	Moderately soluble	High	Yes	0.55
6	Diisobutyl phthalate	C_16_H_22_O_4_	278.34	Moderately soluble	High	Yes	0.55
7	Ethyl salicylate	C_9_H_10_O_3_	166.17	Soluble	High	Yes	0.55
8	Thiophen-2-yl benzoate	C_11_H_8_O_2_S	204.25	Soluble	High	Yes	0.55
9	1-Hexadecanol	C_16_H_34_O	242.44	Moderately soluble	High	Yes	0.55
10	cis-Linoleic acid	C_17_H_30_O_2_	266.4	Moderately soluble	High	Yes	0.85
11	Methyl 9-methyltetradecanoate	C_16_H_32_O_2_	256.42	Moderately soluble	High	Yes	0.55
12	Methyl decanoate	C_11_H_22_O_2_	186.29	Soluble	High	Yes	0.55
13	Methyl dodecanoate	C_13_H_26_O_2_	214.34	Moderately soluble	High	Yes	0.55
14	Methyl linoleate	C_19_H_34_O_2_	294.5	Moderately soluble	High	No	0.55
15	Methyl palmitate	C_17_H_34_O_2_	270.5	Poorly soluble	High	Yes	0.55
16	Methyl tetradecanoate	C_15_H_30_O_2_	242.4	Moderately soluble	High	Yes	0.55
17	Palmitic acid	C_16_H_32_O_2_	256.42	Moderately soluble	High	Yes	0.85
18	Undecylenic acid	C_11_H_20_O_2_	184.27	Soluble	High	Yes	0.85
19	7-O-Methylaloesin	C_20_H_24_O_9_	408.4	Soluble	Low	No	0.55
20	Aloenin	C_19_H_22_O_10_	410.4	Soluble	Low	No	0.55
21	Orcinyl angelate	C_12_H_14_O_2_	206.24	Soluble	High	Yes	0.55
22	(E)-2-Isopropyl-5-methylphenyl 2-methylbut-2-enoate	C_15_H_20_O_2_	232.32	Moderately soluble	High	Yes	0.55
23	(R)-Ar-Turmerone	C_15_H_20_O	216.32	Moderately soluble	High	Yes	0.55
24	Ar-Turmerone	C_15_H_20_O	216.32	Moderately soluble	High	Yes	0.55
25	Carvacrol	C_10_H_14_O	150.22	Soluble	High	Yes	0.55
26	Curlone	C_15_H_22_O	218.33	Soluble	High	Yes	0.55
27	Diosgenin,dehydro	C_27_H_40_O_2_	396.6	Moderately soluble	Low	No	0.55
28	Hinesol	C_15_H_26_O	222.37	Soluble	High	Yes	0.55
29	Nerol	C_10_H_18_O	154.25	Soluble	High	Yes	0.55
30	Phytol	C_20_H_40_O	296.5	Moderately soluble	Low	No	0.55
31	Turmerone	C_15_H_22_O	218.33	Soluble	High	Yes	0.55
32	Heptadecane	C_17_H_36_	240.5	Poorly soluble	Low	No	0.55
33	Pentadecane	C_15_H_32_	212.41	Moderately soluble	Low	No	0.55
34	beta-Sitosterol	C_39_H_50_O	414.7	Poorly soluble	Low	No	0.55
35	1-Heptadecene	C_17_H_34_	238.5	Poorly soluble	Low	No	0.55

Permeability glycoprotein (P-gp) influences the ADMET properties of many xenobiotics (foreign drugs or chemicals), and it is important to investigate the interaction between the transporter protein and the drug molecule^38^. It limits cellular uptake and metabolism of compounds by acting as a unidirectional efflux pump to extrude its substrate from inside to outside of cells^39,40^. From Table 3, it can be seen that all of the phytochemicals except phytol were not substrates of P-glycoprotein, demonstrating their desirable properties as potential therapeutic agents.

**Table 3:** Interaction of Ethiopian indigenous aloes’ phytochemicals with P-glycoprotein and cytochrome P450 isoenzymes.

No	Compound	Pgp substrate	CYP1A2 inhibitor	CYP2C19 inhibitor	CYP2C9 inhibitor	CYP2D6 inhibitor	CYP3A4 inhibitor
1	Aloe-emodin	No	Yes	No	No	No	Yes
2	Homonataloin	No	Yes	No	No	No	Yes
3	Microdontin A	No	No	No	No	No	No
4	Microdontin B	No	No	No	No	No	No
5	Benzyl salicylate	No	Yes	Yes	No	No	No
6	Diisobutyl phthalate	No	Yes	Yes	No	No	No
7	Ethyl salicylate	No	No	No	No	No	No
8	Thiophen-2-yl benzoate	No	Yes	Yes	Yes	No	No
9	1-Hexadecanol	No	Yes	No	No	No	No
10	cis-Linoleic acid	No	Yes	No	Yes	No	No
11	Methyl 9-methyltetradecanoate	No	No	No	Yes	No	No
12	Methyl decanoate	No	No	No	No	No	No
13	Methyl dodecanoate	No	Yes	No	No	No	No
14	Methyl linoleate	No	Yes	No	Yes	No	No
15	Methyl palmitate	No	Yes	No	No	No	No
16	Methyl tetradecanoate	No	Yes	No	No	No	No
17	Palmitic acid	No	Yes	No	Yes	No	No
18	Undecylenic acid	No	No	No	No	No	No
19	7-O-Methylaloesin	No	No	No	No	No	No
20	Aloenin	No	No	No	No	No	No
21	Orcinyl angelate	No	No	No	No	No	No
22	(E)-2-Isopropyl-5-methylphenyl 2-methylbut-2-enoate	No	No	No	No	No	No
23	(R)-Ar-Turmerone	No	No	No	No	No	No
24	Ar-Turmerone	No	No	No	No	No	No
25	Carvacrol	No	Yes	No	No	No	No
26	Curlone	No	No	Yes	Yes	No	No
27	Diosgenin,dehydro	No	No	No	Yes	No	No
28	Hinesol	No	No	No	Yes	No	No
29	Nerol	No	No	No	No	No	No
30	Phytol	Yes	No	No	Yes	No	No
31	Turmerone	No	No	No	Yes	No	No
32	Heptadecane	No	Yes	No	No	No	No
33	Pentadecane	No	Yes	No	No	No	No
34	beta-Sitosterol	No	No	No	No	No	No
35	1-Heptadecene	No	Yes	No	No	No	No

Cytochrome P450s (CYPs) represent a large class of heme-containing enzymes that are necessary for the detoxification of foreign chemicals and the metabolism of drugs. Cytochrome P450 enzymes can be inhibited or induced by drugs, resulting in clinically significant drug-drug interactions that can cause unanticipated adverse reactions or therapeutic failures. Inhibitors block the metabolic activity of one or more CYP450 enzymes whereas inducers increase CYP450 enzyme activity by increasing enzyme synthesis. Hence, cautions should be taken when prescribing a drug known to be a CYP450 inhibitor or inducer. The target drug may need to be substituted or the dose adjusted to account for a potential decrease or increase in metabolism^41^.

### Pharmacophore model-based in silico profiling of phytochemicals from Ethiopian indigenous aloes

The profiling results were shown in two HTML tables designated MoleculeFits and PharmacophoreFits. The two measures utilized to assess the ligand and pharmacophore’s fitness were fit-value and shape similarity. Targets were chosen from the ligand profiler using a fit-value criterion of equal to or higher than 0.5.

Finally, 458 pharmacophore models were mapped. The models belonged to 272 target proteins/genes with unique UniProt-ID. 82 of the targets were related to human, and the CODA^42^ network was employed for further analysis. A complete list of the 1,885 phytochemical-target pairs with target short name, gene and protein IDs, UniProt-ID is presented in (Supplementary Table S1 online).

### Inferring the pharmacological actions using KEGG, GO and network analysis

**Figure 1:**
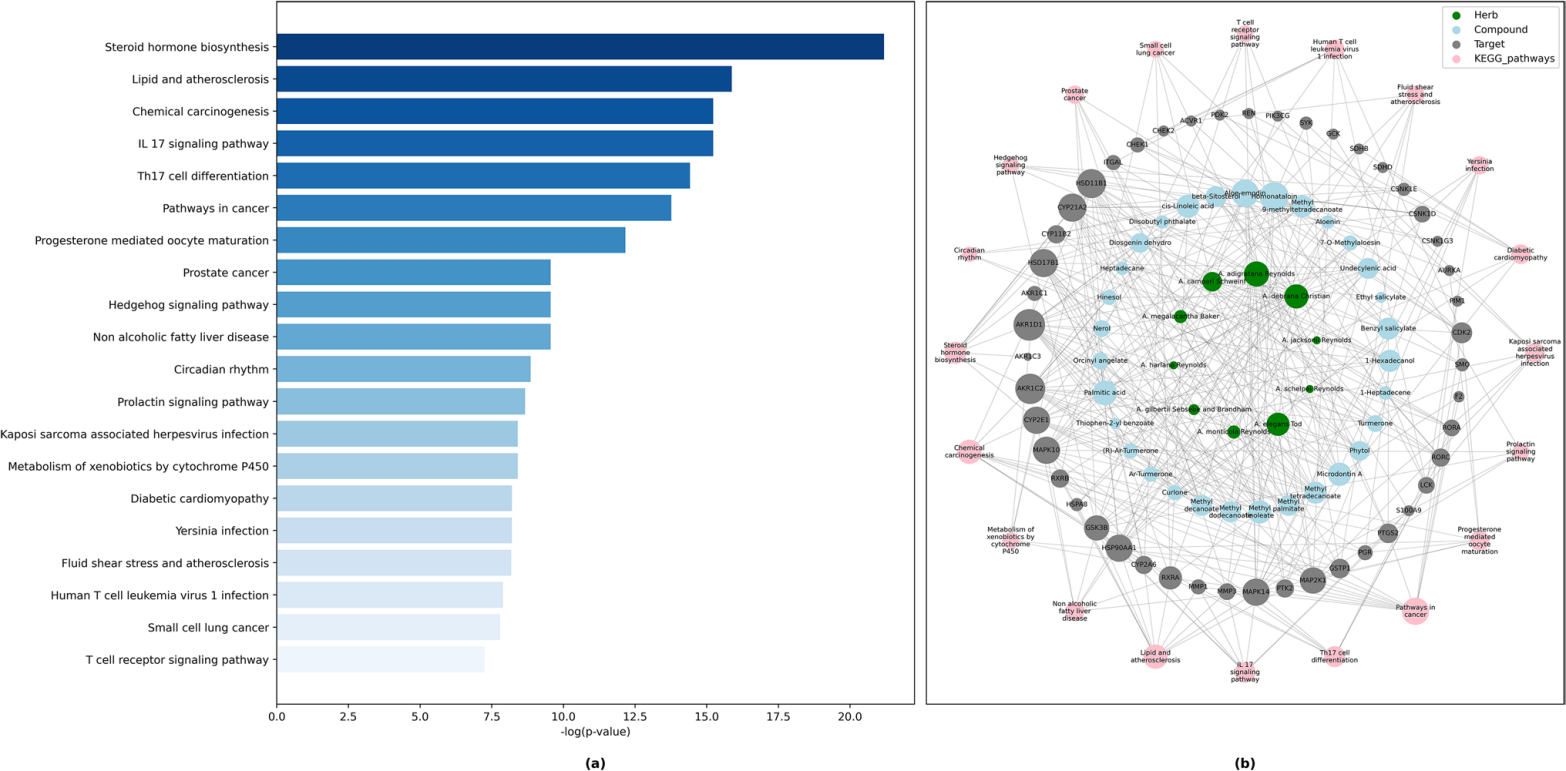
(**a**) Bar graph of KEGG analysis for top 20 enriched pathways. (**b**) Phytochemical-target-pathway network constructed by python NetworkX library illustrated pathways and their associated genes (Adjusted P-value ≤ 0.05).

In this work, a total of 35 compounds found in 10 aloes were successfully mapped to 82 human related targets using pharmacophore models. 207 KEGG pathways^25^ were enriched with the 82 targets of Ethiopian indigenous aloes’ phytochemicals (Supplementary Table S2 online). Based on the adjusted P-value of less than or equal to 0.05, 104 KEGG pathways were filtered. Figure 1a showed top 20 significantly enriched KEGG pathways. A drug-target-pathway network constructed using python NetworkX library version 2.5 illustrated that 47 genes remarkably associated with the top 20 KEGG pathways. Most of the candidates from the network were involved in seven major KEGG pathways, namely metabolism of steroid hormone biosynthesis, lipid and atherosclerosis, chemical carcinogenesis, IL-17 signaling pathway, Th17 cell differentiation and pathways in cancer pathways (Fig. 1b). KEGG enrichment analysis highlighted targets and pathways where the phytochemicals could effectively execute their therapeutic potential. Steroid hormones play an essential role in regulating water and salt balance, metabolism and stress response, and in initiating and maintaining sexual differentiation and reproduction^43^. Previous studies show that aloes can be used as a biological vehicle for delivery of the hormone^44^. Evidence have shown that inflammation plays an essential role in the development, progression, and outcome of atherosclerosis. Owing to their anti-inflammatory properties, aloes may be a good candidate to prevent the progression of atherosclerosis^45^. The phytochemicals found in aloes, especially aloe-emodin, have multiple anti-proliferative and anti-carcinogenic properties in various human cancer cell lines, often affecting multiple important signaling pathways. The most notable effects are inhibition of cell proliferation, cell migration and invasion, cell cycle arrest, induction of cell death, mitochondrial membrane and redox disturbances, and regulation of immune signaling. Aloe-emodin effects are not ubiquitous in all cell lines and vary by cell type^46,47^. Whether looking at aloe as a whole or looking at its compounds, there are various articles that aloes are medicinal plants that have been shown to be beneficial against many types of cancers^47^. However, the precise mechanism of action of aloes’ phytochemicals on underlying molecular targets remains to be investigated.

**Figure 2:**
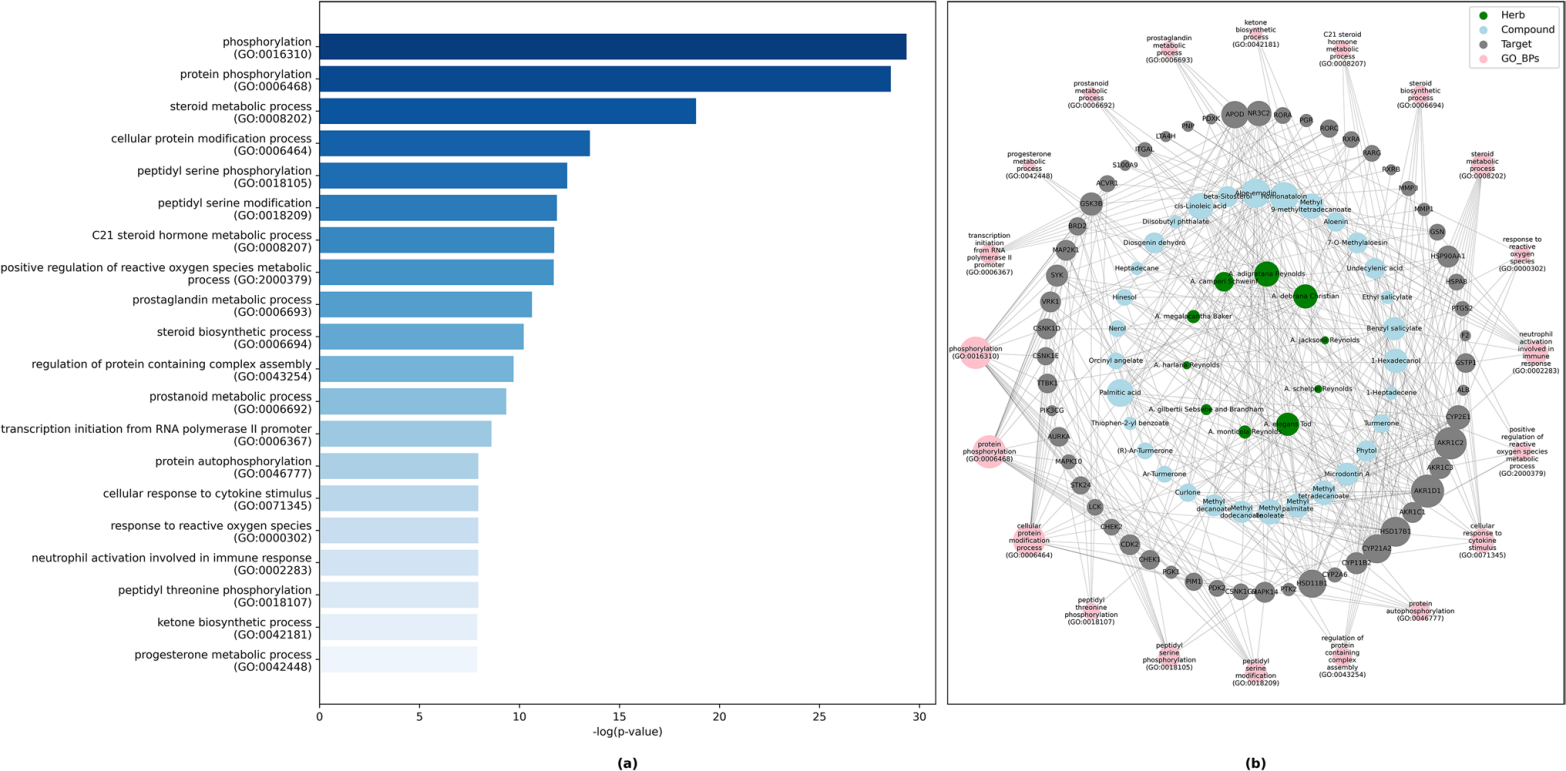
**(a)** Bar graph of KEGG analysis for top 20 enriched biological processes. **(b)** Phytochemical-target-biological process network constructed by python NetworkX library illustrated pathways and their associated genes (Adjusted *P* ≤ 0.05).

GO analysis^48^ is a useful tool for discovering biological processes, cellular components and molecular functions of genes^49^. A total of 1376 biological processes (BPs), 213 molecular functions (MFs), and 101 cellular components (CCs) related to the 82 predicted target proteins/genes were identified (Supplementary Table S2 online). Based on the *P*-value of less than or equal to 0.05, 211 BPs, 58 MFs and 9 CCs were filtered. The functional enrichment analysis of the top 20 BPs demonstrated that most of the targets were correlated with the protein phosphorylation and modification processes, steroid metabolic processes and so on, as shown in Fig. 2a. In addition, a drug-target-biological process network constructed using python NetworkX library version 2.5 illustrated that 55 targets were predominantly enriched with the top 20 BPs (Fig. 2b).

**Figure 3:**
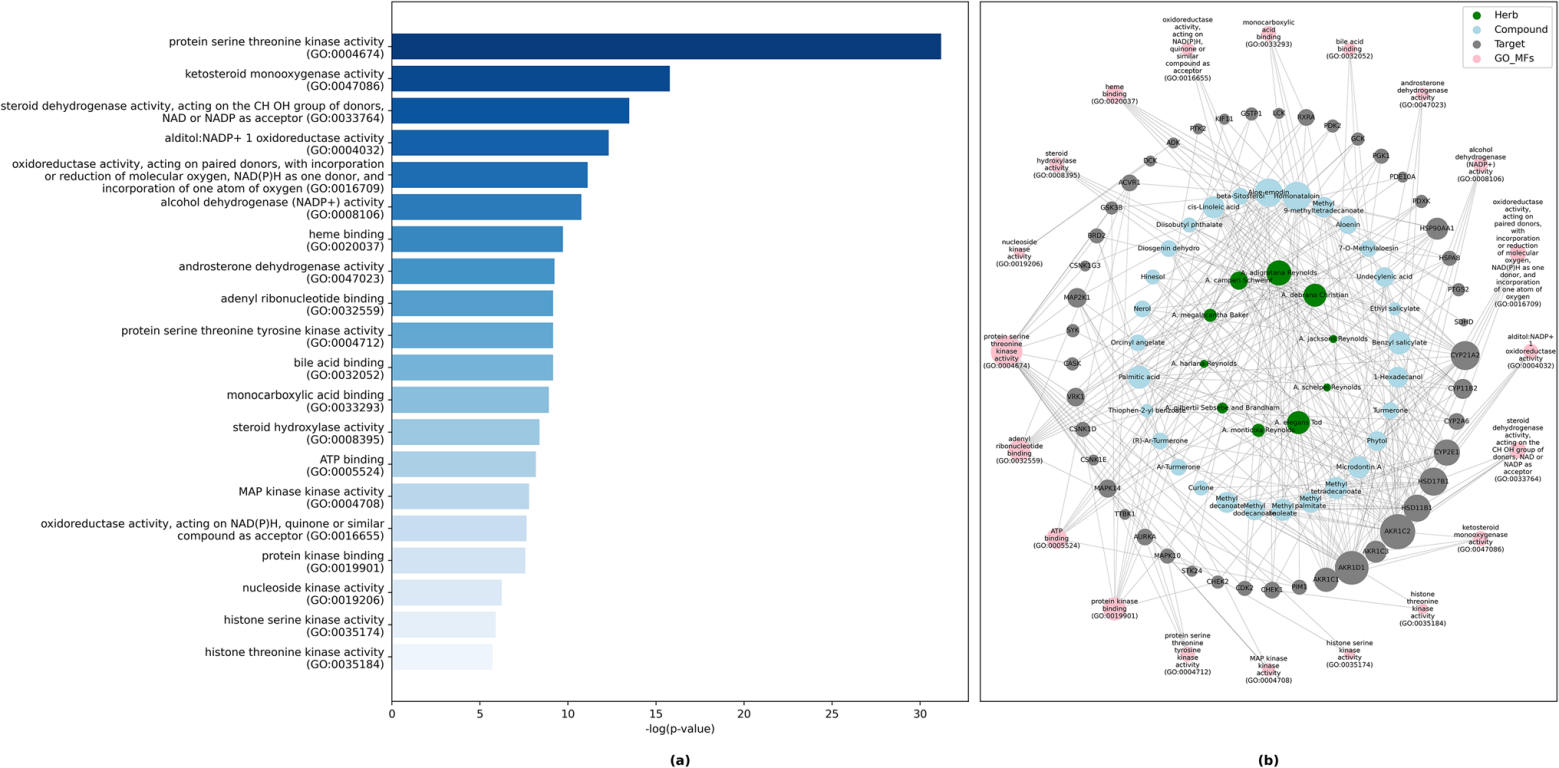
(**a**) Bar graph of KEGG analysis for top 20 enriched molecular functions. (**b**) Phytochemical-target-molecular function network constructed by python NetworkX library illustrated pathways and their associated genes (Adjusted *P* ≤ 0.05).

The top 20 functionally enriched MFs are presented in Fig. 3. Among the major 20 MFs, protein serine/thereonin kinase activity and ketosteroid monooxygenase activity are dominantly expressed by the 55 target genes (Fig. 3a). Figure 3b shows a drug-target-biological process network illustrating that 45 genes were predominantly enriched with the top 20 MFs.

**Figure 4:**
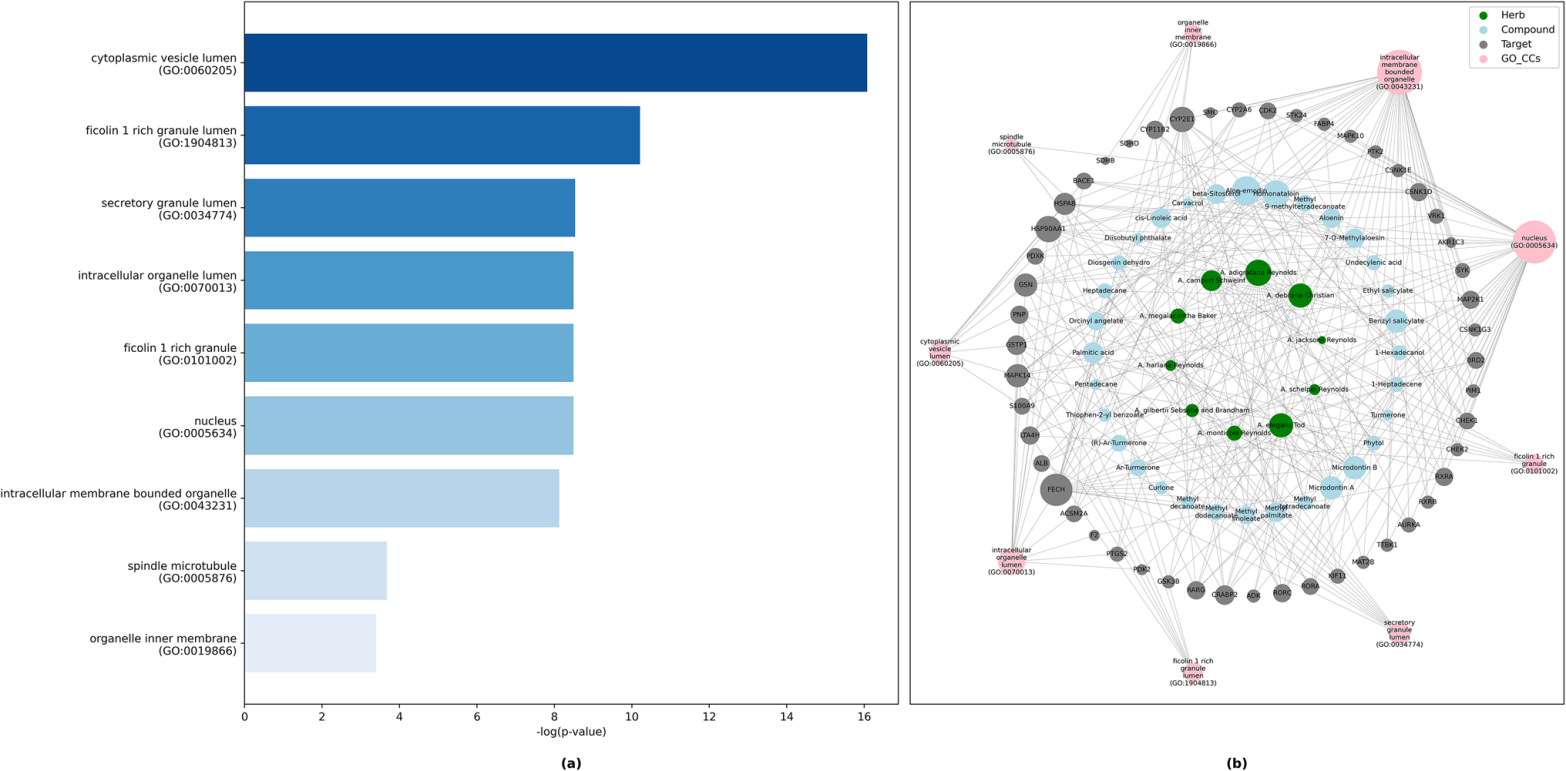
(**a**) Bar graph of KEGG analysis for top 20 enriched cellular components. (**b**) Phytochemical-target- cellular components network constructed by python NetworkX library illustrated pathways and their associated genes (Adjusted *P* ≤ 0.05).

Furthermore, CCs are widely distributed in the lumen of cytoplasmic vesicles, ficolin-1-rich granules, secretory granules and so on (Fig. 4a and b).

In addition, we performed KEGG pathways, GO and network analysis for each of the 10 aloe species used in this study. The figures are presented in Supplementary Figure S1 online. 15 genes related to *A. adigratana* Reynolds’ phytochemicals were enriched in four major KEGG pathways, namely steroid hormone biosynthesis, chemical carcinogenesis, metabolism of xenobiotics by cytochrome p450 and natural killer cell mediated cytotoxicity. The functional enrichment analysis reveals that most of the targets of *A. adigratana* Reynolds’ phytochemicals were related to steroid metabolic and biosynthetic process, progesterone metabolic process, glucocorticoid metabolic and biosynthetic process and positive regulation of reactive oxygen metabolic processes. Moreover, ketosteroid monooxygenase activity, steroid dehydrogenase activity, alditol:NADP + 1-oxidoreductase activity, oxidoreductase activity, and alcohol dehydrogenase (NADP +) activity were the major MFs related to *A. adigratana* Reynolds’ phytochemicals. Similarly, 23 targets were predicted for phytochemicals found in *A. camperi* Schweinf. These targets were enriched in KEGG pathways of steroid hormone biosynthesis, chemical carcinogenesis, Th17 cell differentiation, circadian rhythm, progesterone-mediated oocyte maturation, cellular senescence, p53 signaling pathway, small cell lung cancer, and IL-17 signaling pathway and so on. The main enriched BPs for this aloes’ phytochemicals targets included: steroid metabolic process, steroid biosynthetic process, protein phosphorylation, transcription initiation from RNA polymerase II promoter, phosphorylation, DNA-templated transcription initiation, glucocorticoid biosynthetic process, glucocorticoid metabolic process, and regulation of cellular carbohydrate metabolic process. Furthermore, the main MFs associated with this aloes’ phytochemicals were: protein serine/threonine kinase activity, steroid dehydrogenase activity, heme binding, ketosteroid monooxygenase activity, oxidoreductase activity, steroid hydroxylase activity, and oxysterol binding. In a similar fashion, phytochemicals from other aloe species have related KEGG pathways, BPs and CCs as shown in Supplementary Figure S1 online.

By comparing the top 20 biological processes and pathways enrichment data, 42 targets were found in common and the top ten pathways related to these targets were steroid hormone biosynthesis, lipid and atherosclerosis, chemical carcinogenesis, IL-17 signaling pathway, pathways in cancer, Th17 cell differentiation, progesterone-mediated oocyte maturation, prostate cancer, kaposi sarcoma-associated herpesvirus infection, and human T-cell leukemia virus 1 infection.

### Potential therapeutic activities of Ethiopian indigenous aloes

According to pharmacological profiling, phytochemicals from Ethiopian indigenous aloes were linked to cancers (66 targets), endocrine diseases (16 targets), metabolic diseases (15 targets), immune system diseases (5 targets), nervous system diseases (11 targets), congenital disorders of metabolism (3 targets), musculoskeletal diseases (3 targets), digestive system diseases (3 targets), hematologic diseases (2 targets), infectious diseases (3 targets), mouth and dental diseases (1 targets). Some of these pharmacological activities are related to traditionally reported medicinal uses of the aloes.

From our analysis, we found that around 10 cancer types namely adenocarcinoma, breast carcinoma, glioma, malignant neoplasm of breast, malignant neoplasm of prostate, mammary neoplasms, prostatic neoplasms, squamous cell carcinoma of esophagus, leukemia, and melanoma were related to 65 targets that were linked to 34 compounds from all of the 10 aloe species used in this study. Supplementary Table S3 online provides information on the predicted targets and diseases for each phytochemical found in indigenous Ethiopian aloes.

*A. adigratana* Reynolds phytochemicals were predicted to have therapeutic effect against several cancers (adenocarcinoma, breast carcinoma, malignant neoplasm of prostate, and prostatic neoplasms), endocrine and metabolic diseases (diabetes mellitus, non-insulin-dependent obesity), immune system diseases (colitis, inflammatory bowel diseases and ulcer), digestive system disease (ulcer), and infectious diseases (ulcer and colitis). In northern Ethiopia, *A. adigratana* Reynolds was traditionally used to treat a variety of diseases, including wounds, infections, and inflammation^50^. Previous in vivo and in vitro studies also support the use of *A. adigratana* Reynolds in traditional medicine by confirming its anti-inflammatory properties, which were at least partially related to the presence of aloin A/B and microdontin A/B in the leaves^50^.

*A. camperi* Schweinf. phytochemicals were predicted to have therapeutic effect on cancers (adenocarcinoma, breast carcinoma, malignant neoplasm of breast, malignant neoplasm of prostate, mammary neoplasms, prostatic neoplasms), and endocrine and metabolic diseases (diabetes mellitus- non-insulin-dependent, insulin resistance, morbid obesity, obesity and morbid obesity). Although there is little information on the traditional utilization of the plant, it is claimed to be used for wound healing and malaria treatment^51^.

The potential therapeutic activity of the active phytochemicals inferred in this study should be further investigated using in vivo and in vitro scientific experiments on the predicted diseases.

### Protein–protein interaction (PPI) network, molecular docking and molecular dynamics (MD) simulations

**Figure 5:**
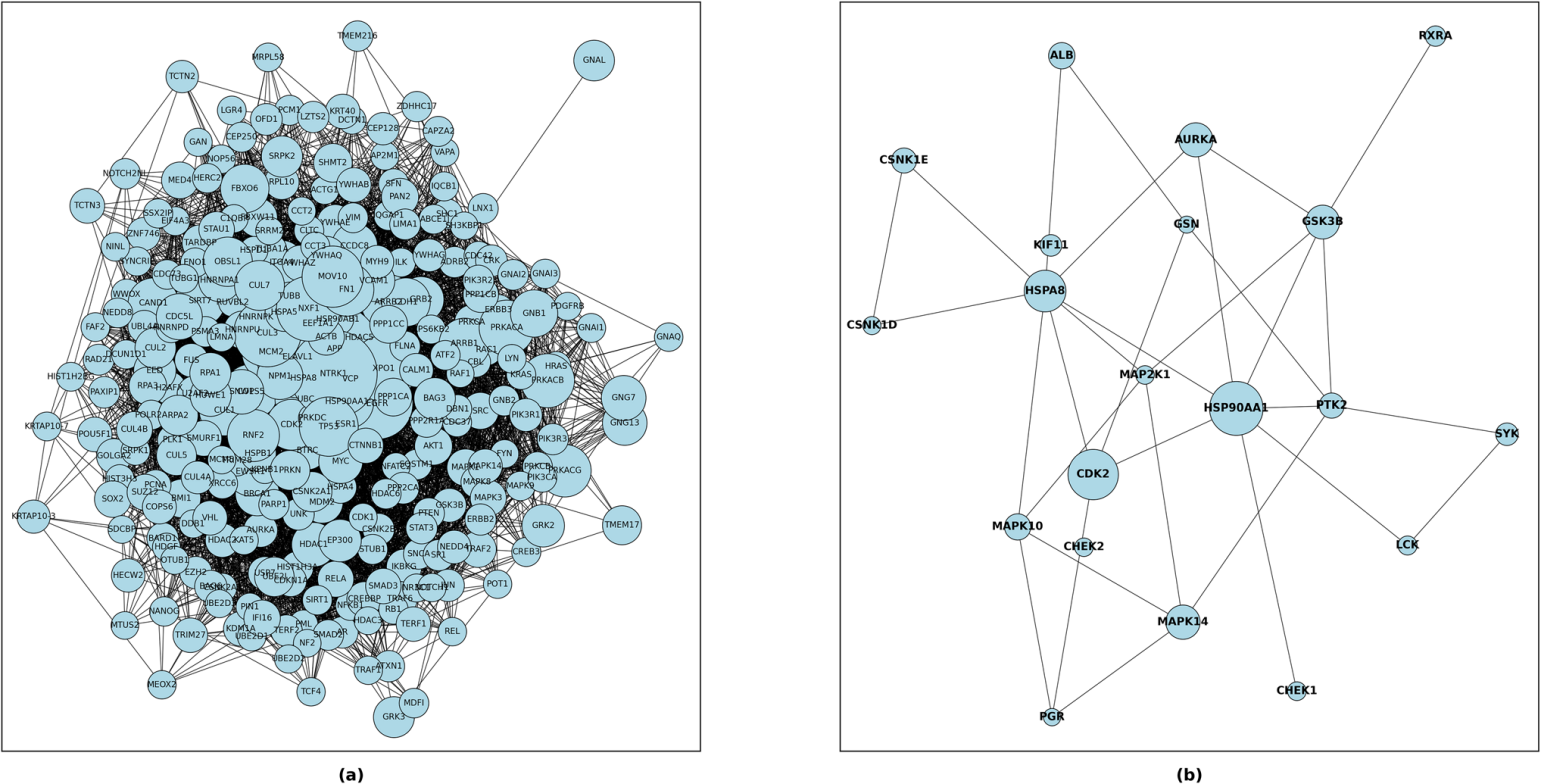
(**a**) PPI network constructed with CODA. (**b**) Network of hub predicted target genes.

**Figure 6:**
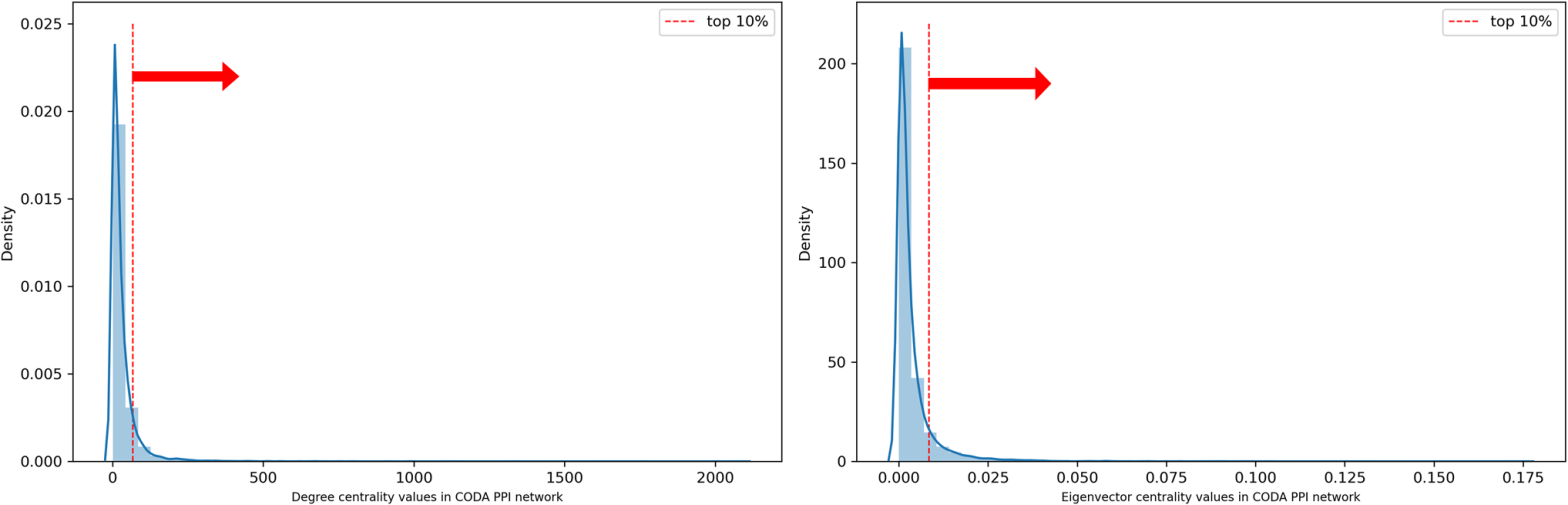
Distribution of centralities of all nodes in protein–protein interaction network. (Left) shows distribution of degree centrality and, (Right) shows distribution of eigenvector centrality.

There were a total 17,358 protein nodes in the CODA PPI network (Fig. 5a) and the distribution of both degree centrality and eigenvector centrality were examined. The degree centrality values of the top 1, 5 and 10% nodes were greater than or equal to 243, 101 and 67 respectively. The eigenvector centrality values were greater than or equal to 0.0313, 0.0138 and 0.008 for 1, 5 and 10% of the nodes respectively. 20 predicted targets (CDK2, HSPAA1, HSPA8, AURKA, MAPK14, GSK3B, MAPK10, PTK2, CSNK1E, SYK, CHEK1, MAP2K1, KIF11, ALB, RXRA, GSN, CHEK2, PGR, CSNK1D, and LSK) were identified to be in the top 10% degree and eigenvector centralities (Fig. 5b). Among them, the targets CDK2, HSPAA1, HSPA8, AURKA, and MAPK14 lies in the top 1% of degree and eigenvector centralities (Fig. 6).

**Figure 7:**
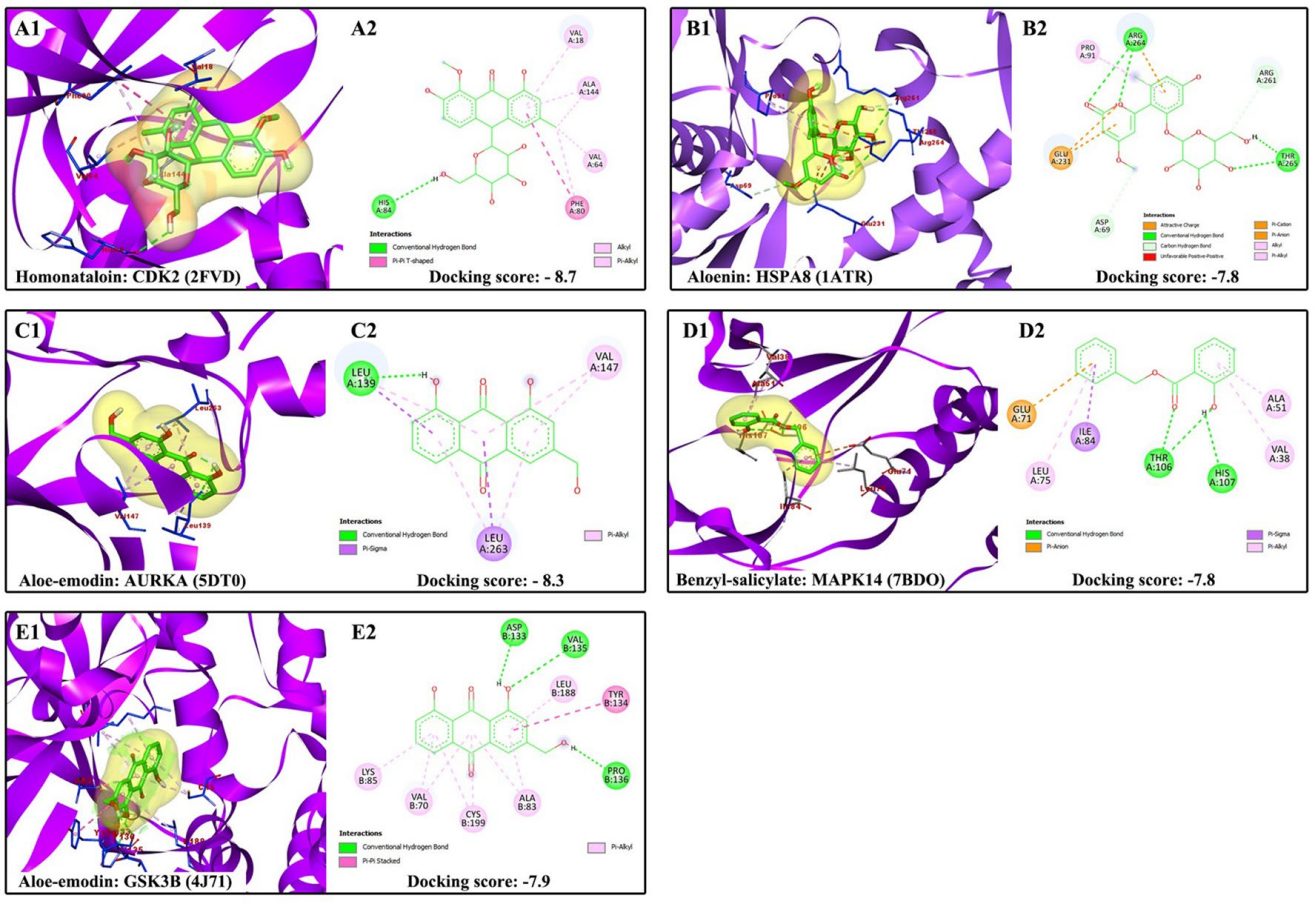
Molecular docking analysis of selected phytochemicals and targets. (**A**1-**E**1) 3D pose views of interaction selected phytochemicals and targets. (**A**2-**E**2) 2D pose views of interaction of selected phytochemicals and targets.

The docking results showed successful binding of the ligands to the target proteins with significantly lower (*p* ≤ 0.05) binding energies compared to the docking scores of random compounds from PubChem to random targets from PDB, confirming the interactions predicted by pharmacophore models in this study. The binding affinity energies, interacting amino acid residues, docking center and docking size between selected phytochemicals and targets are shown in Table 4. Figure 7 shows the 3D and 2D pose views of the interactions between randomly selected phytochemicals from Ethiopian indigenous aloes and hub gene/protein in PPI network of the predicted targets.

**Table 4:** Binding affinity energies, interacting amino acid residues, docking center and docking size between selected phytochemicals and targets.

Phytochemicals	Target (PDB ID)	Docking score (kcal/mol)	Interacting amino acid residues	Docking center (x, y, z)	Docking size (x, y, z)
Homonataloin	CDK2 (2FVD)	− 8.7	ILE10, GLU12, GLY13, VAL18, ALA31, LYS33, VAL64, PHE80, GLU81, PHE82, LEU83, HIS84, GLN85, ASP86, GLN131, ASN132, LEU134, ALA144, ASP145, GLU162	− 4, 30, 10	21, 21, 21
Aloenin	HSPA8 (1ATR)	− 7.8	TYR41, LYS56, VAL59, PRO63, PHE68, ASP69, HIS89, PRO91, GLU231, ASN235, ARG261, ARG262, ARG264, THR265, GLU268	14, 32, 19	30, 35, 30
Aloe-emodin	AURKA (5DT0)	− 8.3	LEU139, GLY140, VAL147, ALA160, LYS162, LEU194, LEU210, GLU211, TYR212, ALA213, GLY216, THR217, GLU260, LEU263, ALA273, ASP274	− 6, − 37, 4	20, 28, 20
Benzyl-salicylate	MAPK14 (7BDO)	− 7.8	VAL30, TYR35, VAL38, ALA51, LYS53, GLU71, LEU75, ILE84, LEU104, THR106, HIS107, LEU108, MET109, LEU167, ASP168, PHE169	18, 32, 18	20, 30, 20
Aloe-emodin	GSK3B (4J71)	− 7.9	ILE62, GLY63, VAL70, ALA83, LYS85, GLU97, VAL110, LEU132, ASP133, TYR134, VAL135, PRO136, LEU188, CYS199, ASP200, PHE201	− 40, 7, 43	20, 20, 20

**Figure 8:**
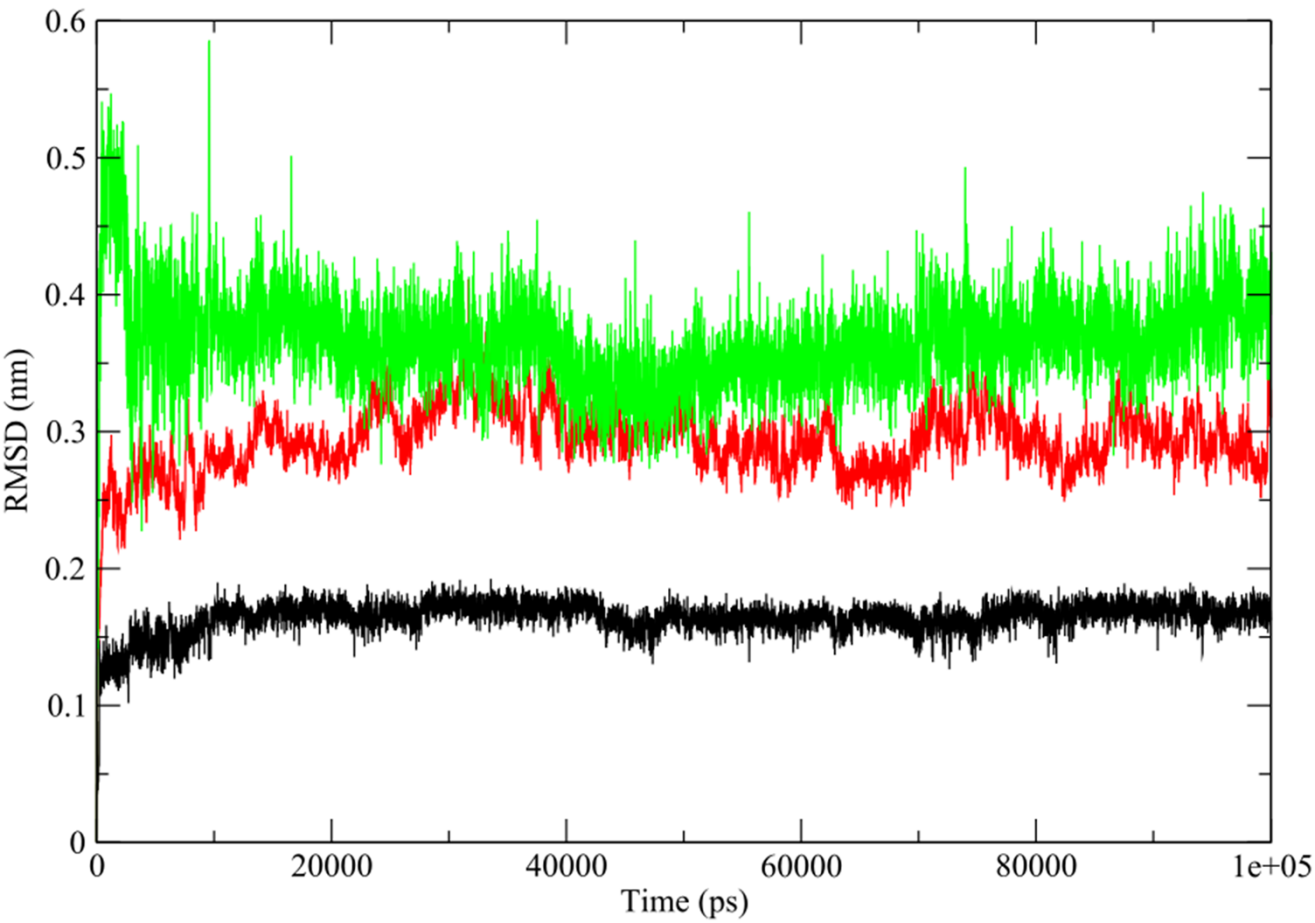
RMSD plots of target proteins (CDK2)—ligand (homonataloin) complex over 100 ns MD simulation.

According to docking analysis, the ligand homonataloin was selected for MD simulations as it has relatively minimum binding energy score (− 8.7 kcal/mol) with target protein CDK2. MD trajectories over 100 ns for the protein—ligand complex was analyzed to investigate the stability of the proteins with bound ligand using root-mean-square deviation (RMSD) parameter. In order to examine the conformational variation of target protein upon interaction with ligand, RMSD for protein—ligand complex, ligand and target protein with respect to the starting structure was calculated and depicted as shown in Fig. 8. It is clearly shown that RMSD of the atoms of protein – ligand complex, protein and ligand have the same pattern of fluctuation with a steady increase in the initial 0.25 ns followed by stability until the end of the simulation. The RMSD of the protein (Fig. 8, red) is slightly higher than that of the ligand (Fig. 8, black) during the course of simulation. Furthermore, the RMSD of the protein—ligand complex (Fig. 8, green) is higher than that of the protein and ligand indicating that there was a conformational change of protein in the complex with the ligand binding. Interestingly, the protein—ligand complex exhibited a very low fluctuations during the simulation period indicating that the complex is stable with the ligand remaining fit in the binding pocket of the receptor (see Supplementary Video S1 online).

**Figure 9:**
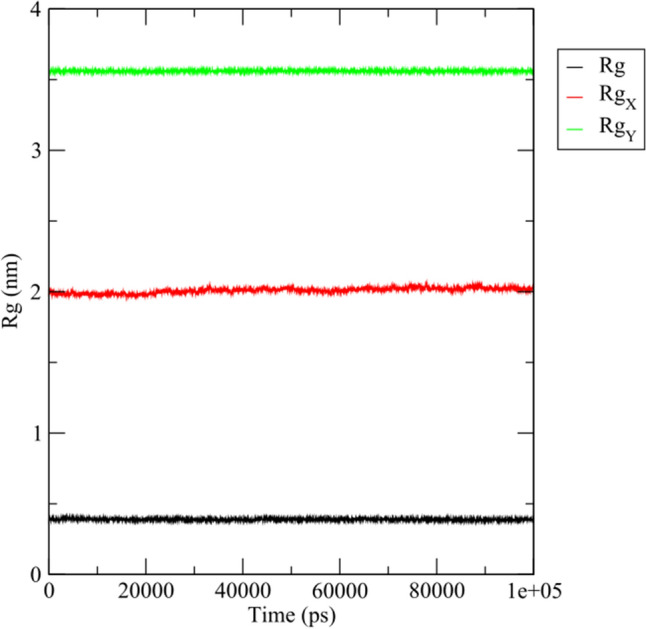
Radius of gyration, Rg (nm) plots of the ligand (Rg), target protein (Rg_X_) and protein–ligand complex (Rg_Y_) over 100 ns simulation run.

An excellent analysis of the dynamics of protein collapse is provided by the time development of the radius of gyration (Rg)^52^. To determine whether the protein was compact, the systems' gyration radii were measured, and a graph showing how they are related to the simulation time was created^53^ as shown in Fig. 9. Similar to RMSD plots, Rg of the ligand, protein and the protein – ligand complex system did not change significantly throughout the simulation indicating that the binding region shows little influence on their structures during the simulation period.

## Methods

**Figure 10:**
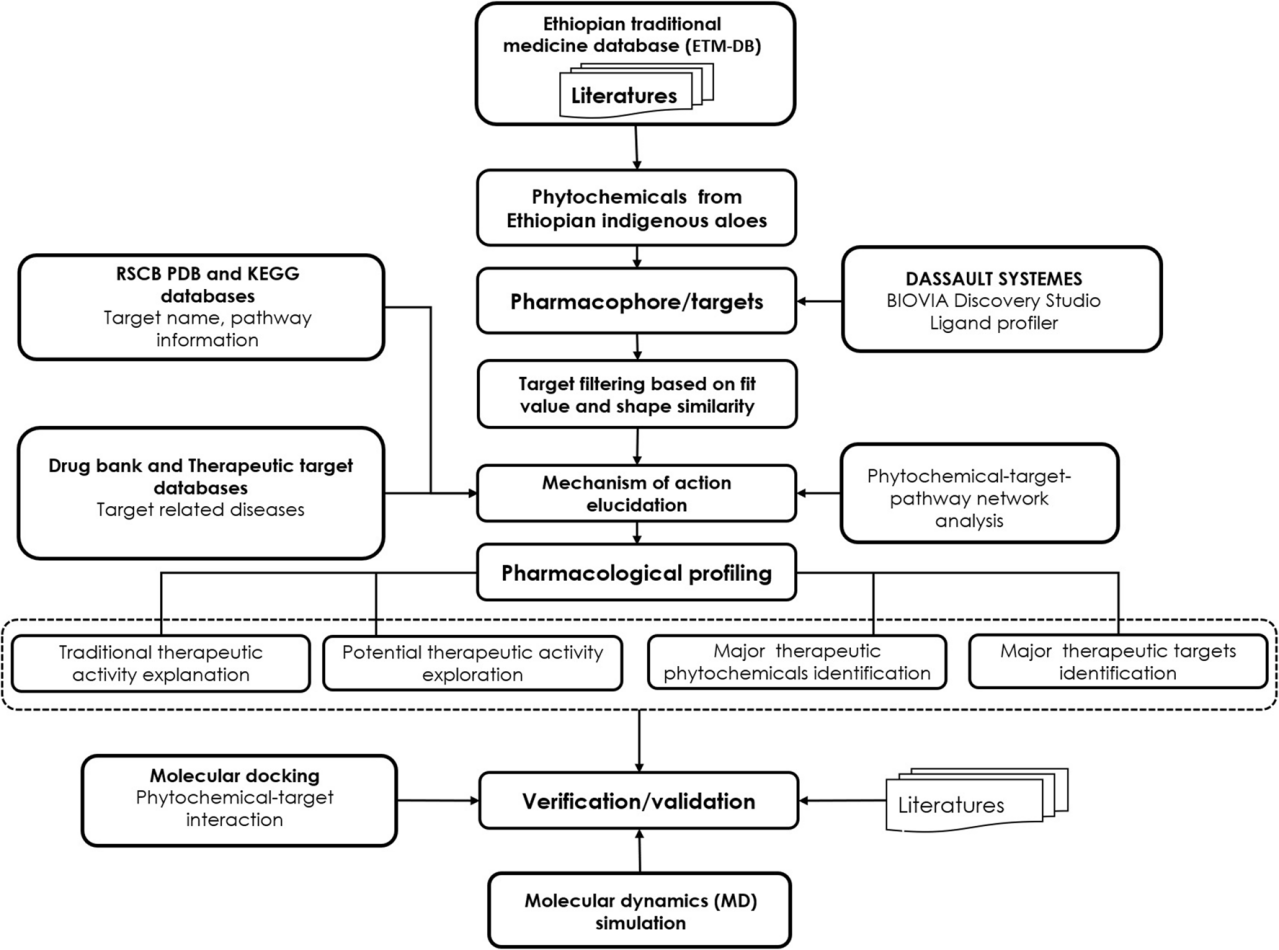
The workflow used in this study.

In this study, pharmacophore model-based ligand profiling was used in target fishing to predict potential targets of compounds from Ethiopian indigenous aloes. Figure 10 shows the workflow of this study. First, Ethiopian indigenous aloes were identified through literature searches and their phytochemicals (compounds) were manually compiled from literatures and integrated Ethiopian traditional herbal medicine and phytochemicals database (ETM-DB)^54^. The three-dimensional (3D) structure data files (SDF) of the phytochemicals were collected from PubChem database^55^. The physicochemical properties of the phytochemicals were retrieved from PubChem database and SwissADME webserver^29^. The phytochemicals were filtered for toxicity using admetSAR. Then, we used PharmaDB pharmacophore and target databases of Discovery Studio^56^ to perform target fishing for the phytochemicals. The hit pharmacophore models were picked out according to the threshold of a predetermined fit value (0.5). After analysis of the hit targets and their associated pathways and diseases, as well as the interactions between the phytochemicals and the targets, an action network of Ethiopian indigenous aloes phytochemicals was constructed. Furthermore, Molecular docking was carried out to refine the binding modes and to verify the findings. Finally, molecular dynamics (MD) simulation was performed for the best binding interaction of the protein—ligand complex.

### Collection of phytochemical constituents of Ethiopian indigenous aloes

**Figure 11:**
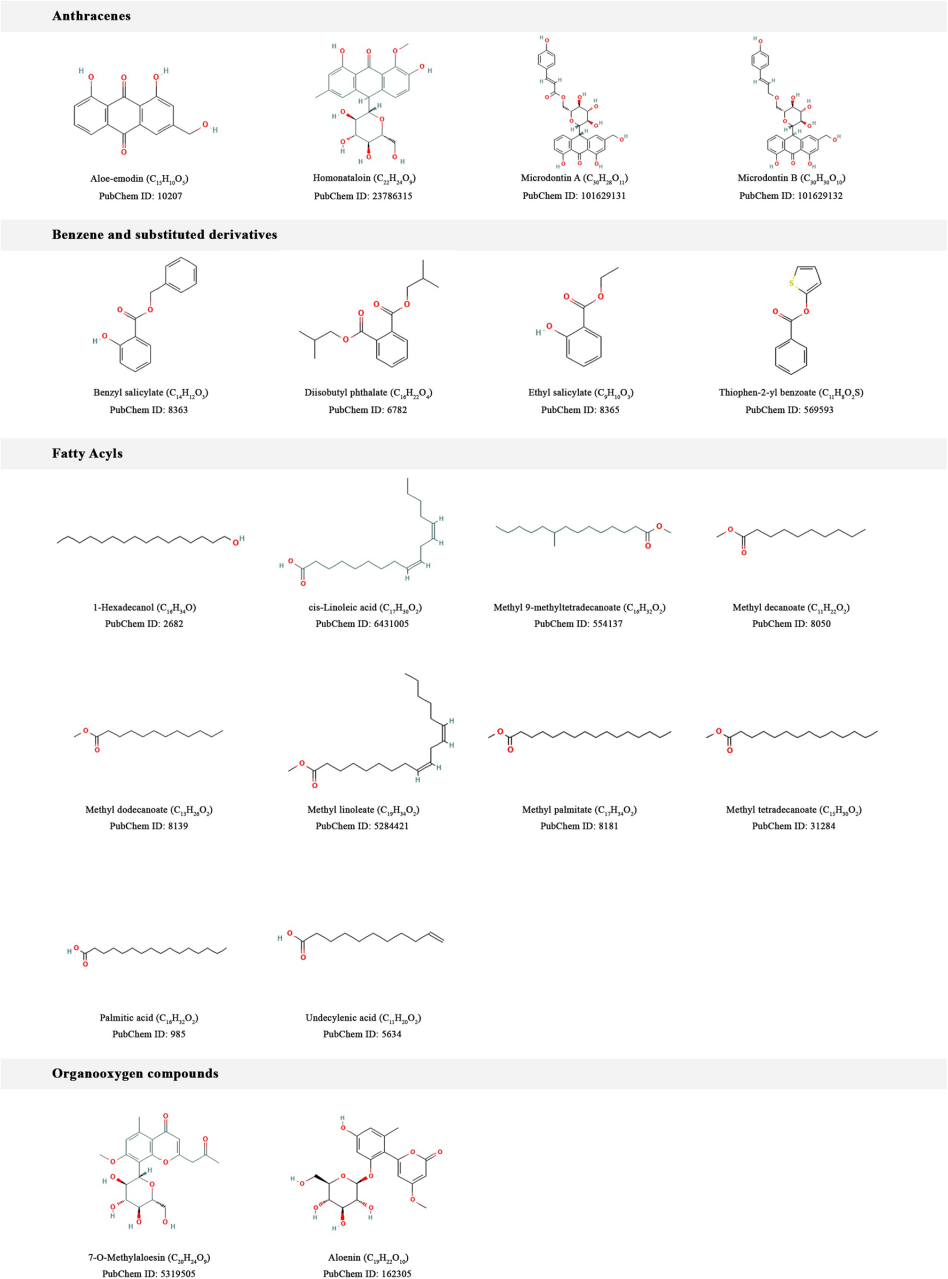

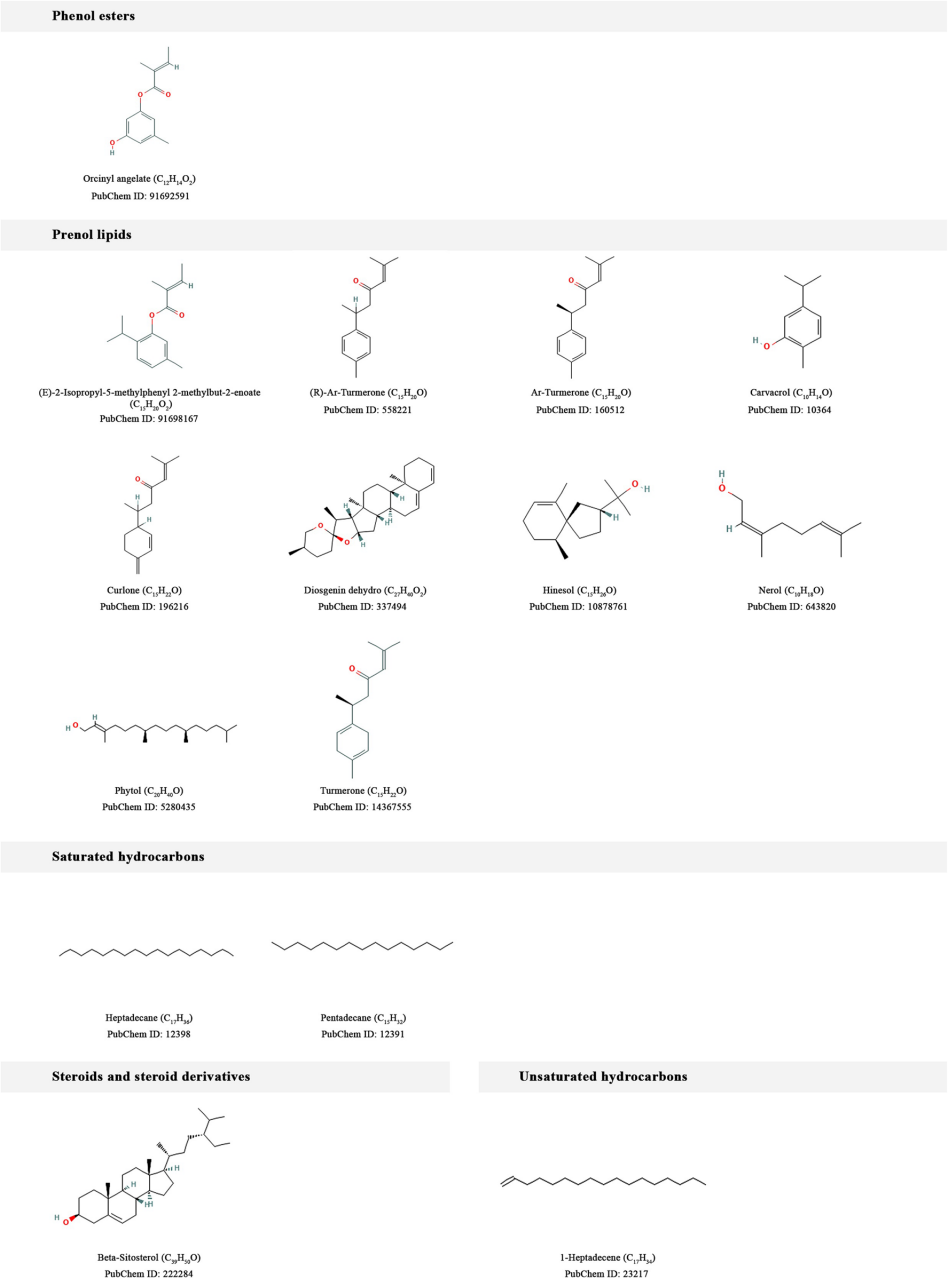
2D chemical structures and compound classes of phytochemical constituents of Ethiopian indigenous aloes whose human targets were successfully predicted using pharmacophore models.

The phytochemical constituents of Ethiopian indigenous aloes were collected from our own database^54^ and the literature. Accordingly, we compiled 35 compounds from 10 Ethiopian indigenous aloes after removing potentially toxic compounds and evaluating their ADMET and drug-likeness properties. And then, targets were successfully predicted for the phytochemicals using computational target fishing. All 35 phytochemicals’ 2D chemical structures and compound classes are listed in Fig. 11. These 35 phytochemicals were classified using ClassyFire (automated chemical classification with a comprehensive, computable taxonomy)^57^ into 9 compound classes as anthracenes, benzene and substituted derivatives, fatty acyls, organooxygen compounds, phenol esters, prenol lipids, saturated hydrocarbons, steroids and steroid derivatives, and unsaturated hydrocarbons.

### Evaluation of physicochemical, drug-likeness and ADMET properties of phytochemicals from Ethiopian indigenous aloes

Physicochemical properties of the phytochemicals were retrieved from PubChem database^28^ and SwissADME webserver^29^. SwissADME is a free web tool to evaluate pharmacokinetics, drug-likeness and medicinal chemistry friendliness of small molecules. We used PubChemPy—a python library used to interact with PubChem database to retrieve information related to phytochemicals from PubChem database.

The drug-likeness properties of the phytochemicals were predicted using the physicochemical properties. Drug-likeness is related to molecular properties affecting pharmacodynamics and pharmacokinetics of the molecules^58^. These molecular properties are related to some basic structural or physicochemical properties such as log *p* (partition coefficient), molecular weight (MW), topological polar surface area (TPSA), hydrogen bond acceptors and donors count in a molecule. There are several sets of criteria to select ligands for a potential drug. Most notably, Lipinski^30^ identified common properties that are frequently observed in approved compounds. The authors presented a “rule of 5” for drug-like molecules (less than 5 hydrogen bond donors, less than 10 hydrogen bond acceptors, less than 500 Daltons (g/mol) molecular weight, and less than 5 log *p* (partition coefficient between organic and aqueous phases)). In addition, Veber’s rule^31^ suggested that compounds which meet only the two criteria of 10 or fewer rotatable bonds and polar surface area equal to or less than 140 Å^2^ have a high probability of good oral bioavailability in rat. However, many of the most successful drugs do not fit these guidelines, and care should be taken in the application of these guidelines^32^. In addition, most of compounds from natural products do not always follow the rules which are commonly applied in modern drug design including Lipinski's “Rule of Five ”^21^.We used compounds with two or more Rule of 5 violations which are used in drug development by medicinal chemists and industries^59^ as a reference for drug-likeness evaluation of the phytochemicals in this research.

ADMET properties were predicted from admetSAR web server^27^ from the compounds’ Simplified Molecular Input Line Entry System (SMILES) string as input using BeautifulSoup (a Python library for pulling data out of HTML and XML file). We removed potentially toxic compounds that were AMES mutagens, carcinogens and human Ether-a-go-go-Related gene (hERG) inhibitors. The inhibition of the human ether-a-go-go (hERG) ion channel may cause cardiotoxicity and hence, it is necessary to screen all potential drug candidates for risk against blocking the hERG channel^60^. The Ames test is a sensitive tool in screening for potential genotoxic carcinogens^61^.

### Pharmacophore model-based in silico profiling of phytochemicals from Ethiopian indigenous aloes

A pharmacophore model represents a target-ligand binding site that triggers a desired pharmacological effect^16^. A pharmacophore models used in this study were derived from protein–ligand 3D complex structures as well as structural data on small bioactive organic molecules^62^. Pharmacophores can be used as low-dimensional targets in target prediction tasks^63^. We performed pharmacophore-based prediction of the potential targets of aloes’ phytochemicals using BIOVIA Discovery Studio^56^. Discovery Studio is an interactive computational chemistry and molecular modeling package with a broad variety of features for modeling and simulation^56^. We used “Ligand Profiler” protocol which is an automated ligand profiling in BIOVIA Discovery Studio. The BIOVIA Discovery Studio provides automated pharmacophore-based activity profiling and reporting^16^. Discovery Studio 4.5 is equipped with PharmaDB pharmacophore database which contains about 68,000 pharmacophores derived from 8000 protein–ligand complexes from the sc-PDB dataset, which itself is a collection of 3D structures of binding site found in PDB^64^. The binding sites were extracted from crystal structures in which a complex between a protein cavity and a small molecule ligand could be identified^15^.

As for parameters setting during ligand profiling with Input PharmaDB Pharmacophores, all the pharmacophore models with the shape of the binding pocket were selected for the virtual screening with default settings. The RIGID mode was used as the molecular mapping algorithm. A conformer database (3D SDF file format) was generated using BEST settings of the Conformation Generator component for each query ligand. All molecular features were allowed while mapping these ligands to the pharmacophore models to increase selectivity. The minimal interfeature distance was set at 0.5 Å. For every query ligand, targets were ranked by decreasing fit-value. The targets were nominated according to their UniProt ID. UniProt is an abbreviation for the Universal Protein Database program, which consists of the Swiss-Prot, TrEMBL and PIR-PSD databases and is the largest database containing the most informative data resources and protein structures^65^. Further information about the protein including the protein name, gene name, organism, sequence information, taxonomy, family and domains, and related molecular functions and biological processes can be obtained using the UniProt ID from UniProt website (http://www.uniprot.org/). This database can also link to other databases by searching based on the PDB ID, KEGG ID and other information. In this work, for each target, the target name, UniProt entry name and UniProt ID information were collected from the PDB^64^ and UniProt databases^65^.

### Inferring the pharmacological actions using KEGG, GO and network analysis

To investigate the therapeutic effect of Ethiopian indigenous aloes, Kyoto Encyclopedia of Genes and Genomes (KEGG) pathways and Gene ontology (GO) of the 82 predicted targets were analyzed. In order to identify the complex relationship between compounds, targets, pathways and biological processes, we conducted enrichment test by using enrichr function in gseapy python library with internal gene set named ‘KEGG_2021_Human’. Network analysis in the Context-Oriented Directed Associations (CODA)^42^ were performed to obtain diseases related to each predicted targets. Network propagation was conducted with Random-Walk with Restart (RWR) algorithm in the heterogeneous biological networks of CODA. CODA is an integrated database which contains diverse types of biological interactions from molecular level to phenotypic level. We constructed a heterogeneous network using all types of biological interactions in CODA to see converging information flow into disease nodes in the network starting from the targets. After running the algorithm, we ranked disease nodes in the network by their RWR scores. Then the disease was mapped to high-level disease category using KEGG Application Programming Interface (API)—a REST-style API to the KEGG database resource.

### Protein–protein interaction (PPI) network, molecular docking and molecular dynamics (MD) simulations

A protein–protein interaction (PPI) network was constructed from CODA^42^, which contains a total of 17,358 protein nodes. Among molecular relations in the CODA, proteins with both left and right entity type of gene product were selected. Then all the nodes were represented with gene symbol for better interpretation. To get hub targets in the PPI network, two types of network centralities (degree centrality and eigenvector centrality) were used. There are also another two more representative network centrality measures such as closeness centrality and betweenness centrality. However, closeness and betweenness centrality were not considered in this study because they focus on the overall robustness in the network than relationship between neighboring nodes^66^. Because of this, we consider hub targets as the nodes within the top 10% degree and eigenvector centrality values in the PPI network.

Additionally, molecular docking studies were performed to confirm the protein–ligand interaction that the pharmacophore models predicted. Blind docking was used for the detection of possible binding sites and modes of peptide ligands by scanning the entire surface of protein targets^67^. In this study, we employed blind docking since we do not have information regarding the binding mode of the phytochemicals with the predicted targets. The PDB files of five randomly chosen proteins from hub proteins in PPI network (obtained from CODA network analysis) were retrieved from Protein Data Bank (PDB), and the ligands’ 3D SDF file was obtained from PubChem database. The molecular docking was done using the CB-Dock2 web server (http://clab.labshare.cn/cb-dock/php/index.php), which is a server for protein–ligand blind docking, integrating cavity detection, docking and homologous template matching. CB-Dock automatically identifies the binding sites, calculates the center and size, customizes the docking box size according to the query ligand and then perform the molecular docking with AutoDock Vina^68^. Then, the 3D and 2D poses of the protein—ligand complexes were analyzed and visualized using Discovery Studio Visualizer.

Furthermore, we compared the docking score of our selected target and protein with the docking scores of random compounds from PubChem docked with random targets from PDB using one-sample, one-sided t-test to statistically show that the bindings were significant (*P* ≤ 0.05).

Among the selected phytochemicals for molecular docking, the ligand homonataloin displayed the best binding interaction and free energy with the Cyclin Dependent Kinase 2 (CDK2) target protein. Accordingly, it was chosen to be subjected to 100 nanoseconds (ns) molecular dynamics (MD) simulation against the target protein to confirm its stable binding affinity. MD simulations were performed using GROningen MAchine for Chemical Simulations (GROMACS) 2021.4 software package^69^ where the CHARMM27 all-atom forcefield^70^ was used for protein topology preparation and the official SwissParam (https://www.swissparam.ch/)^71^ for ligand topology preparation. The solvation method used was a triclinic box of default Simple Point Charge (SPC) water model where explicit solvent periodic boundary conditions were applied. Charge neutralization using sodium and chloride ions was carried out for the solvated complexes. The systems were subjected to energy minimization to resolve any steric clashes or inappropriate geometry employing the steepest descent method through 5000 steps. System equilibration was also manipulated to ensure a reasonable starting structure using NVT; equilibration under constant number of particles, volume, and temperature (NVT) for 100 picoseconds (ps) using a Berendsen thermostat^72^. Also, re-equilibration was performed for another 100 ps under constant pressure (Isothermal-isobaric (NPT) ensemble) using the Parrinello-Rahman barostat using a time step of 2 femtoseconds (fs) for each equilibration round^73^. Finally, MD production phase was carried out for 100 ns using a time step of 2 fs at a constant temperature of 300 K and constant pressure of 1 atm. For MD trajectories analysis, the complex was re-centered and re-wrapped within the unit cells using the “trjconv” function of GROMACS. Then, the trajectories were analyzed using RMSD of the protein, ligand and protein–ligand complex referenced to its initial position and the radius of gyration (Rg). GRaphing and Advanced Computation and Exploration of data (Grace) tool was used for data plotting (https://plasma-gate.weizmann.ac.il/Grace/).

### Computation environment

The BIOVIA Discovery Studio software program were installed on Windows 10. Profiling of the phytochemicals were carried out in an automatic fashion on a Windows 10 computer (Intel Core i5-4690 CPU @ 3.50 GHz, 3501 MHz, 4 Core(s), and 4 Logical Processor(s)). The configuration of the RAM was: 4 slots, 3 of them each having 8 GB RAM and 1 slot 4 GB RAM giving a combined 28 GB, DDR3 and speed of 1600 MHz. Network analysis (Random-Walk with Restart algorithm), KEGG pathway enrichment and GO analysis were performed on a server CentOS computer, Intel Xeon CPU E5-2680 v3 @ 2.50 GHz, 48 cores. The configuration of the RAM was: 16 slots with 32 GB RAM, DDR4. MD simulation was done using: Linux system (Ubuntu 2021.4–2) computer (Intel Core i7-10,700 CPU @ 2.90 GHz × 16, Mesa Intel UHD Graphics 630 (CML GT2) / llvmpipe (LLVM 13.0.1, 256 bits, 64.0 GB RAM)).

## Conclusions

In silico profiling provides a rapid approach for identification of active compounds, prediction of bioactivities of compounds, and to guide the rationalization of the use of traditional medicines. Computational target fishing is a new approach in pharmaceutical research that helps to examine the relationship between drugs and diseases. Using the admetSAR and SwissADME online servers, the ADMET and drug-likeness characteristics of phytochemicals from indigenous aloes in Ethiopia were assessed. Then, using in silico pharmacophore models, potential therapeutic targets were predicted and a wide range of associated therapeutic potential was revealed.

The findings suggested that the phytochemicals in indigenous Ethiopian aloes are potential therapeutic molecules with 82 associated targets that were enriched in a number of KEGG pathways and GO terms. The results demonstrated that the phytochemicals have a broad range of therapeutic targets and the potential to regulate important biological pathways, such as the metabolism of steroid hormone biosynthesis, lipid and atherosclerosis, chemical carcinogenesis, IL-17 signaling pathway, Th17 cell differentiation, and pathways in cancer, among others. Furthermore, this study revealed that the phytochemicals may be used as a potential therapeutic agent for a number of cancers, and diseases of the endocrine, metabolic, immune, nervous, digestive, musculoskeletal, and hematologic systems, as well as congenital disorder of metabolism, infectious diseases, and mouth and dental diseases. Particularly, the aloe species *A. debrana* Christian and *A. megalacantha* Baker were linked cancer and endocrine disease; *A. gilbertii* Sebsebe and Brandham, *A. monticola* Reynolds, and *A. schelpei* Reynolds were linked with congenital disorders of metabolism; *A. adigratana* Reynolds and *A. elegans* Tod were related to digestive system disease; and so forth. This information indicated that these aloes were predicted to be effective in treating the aforementioned diseases. In order to confirm these effects, the phytochemicals’ pharmacological effects should be applied to further controlled experimental research.

## Supplementary Information

Supplementary Information 1.

Supplementary Information 2.

Supplementary Information 3.

Supplementary Information 4.

Supplementary Information 5.

Supplementary Video 1.

## Data Availability

All relevant data are within the paper and supporting information files.
