# Supplementary material for: In silico activity and ADMET profiling of phytochemicals from Ethiopian indigenous aloes using pharmacophore models
Source: Sci Rep. 2022 Dec 23;12:22221. doi: 10.1038/s41598-022-26446-x (PMC9789083; doi:10.1038/s41598-022-26446-x)
Supplement: Supplementary file 1 — Supplementary Information 1. [file 41598_2022_26446_MOESM1_ESM.docx]

**Supplementary Video S1.** Movie of the MD trajectory showing a view of the binding interface between ligand homonataloin and target CDK2
