# Supplementary material for: In silico activity and ADMET profiling of phytochemicals from Ethiopian indigenous aloes using pharmacophore models
Source: Sci Rep. 2022 Dec 23;12:22221. doi: 10.1038/s41598-022-26446-x (PMC9789083; doi:10.1038/s41598-022-26446-x)

In silico activity and ADMET profiling of phytochemicals from Ethiopian indigenous aloes using Pharmacophore models

Lemessa Etana Bultum[1,2,3], Gemechu Bekele Tolossa[2,4], Gwangmin Kim[1,2], Ohhyeon Kwon[1,2] and Doheon Lee[1,2*]

[1]Department of Bio and Brain Engineering, Korea Advanced Institute of Science and Technology (KAIST), 291Daehak-ro, Daejeon 34141, South Korea.
[2]Bio-Synergy Research Center, 291Daehak-ro, 34141 Daejeon, South Korea.

[3]Department of Applied Bioscience, Dong-A University, Busan 49315, South Korea

[4]Department of Neuroscience, Washington University School of Medicine, St. Louis, MO 63110, USA.

*Corresponding author E-mail: dhlee@kaist.ac.kr (DL)

Figures S1. KEGG pathways, GO and network analysis for each of the 10 aloe species used in this study. KEGG pathways and GO analysis for biological processes, molecular functions, and cellular components for all the ten aloe species alongside network diagrams

A. adigratana Reynolds

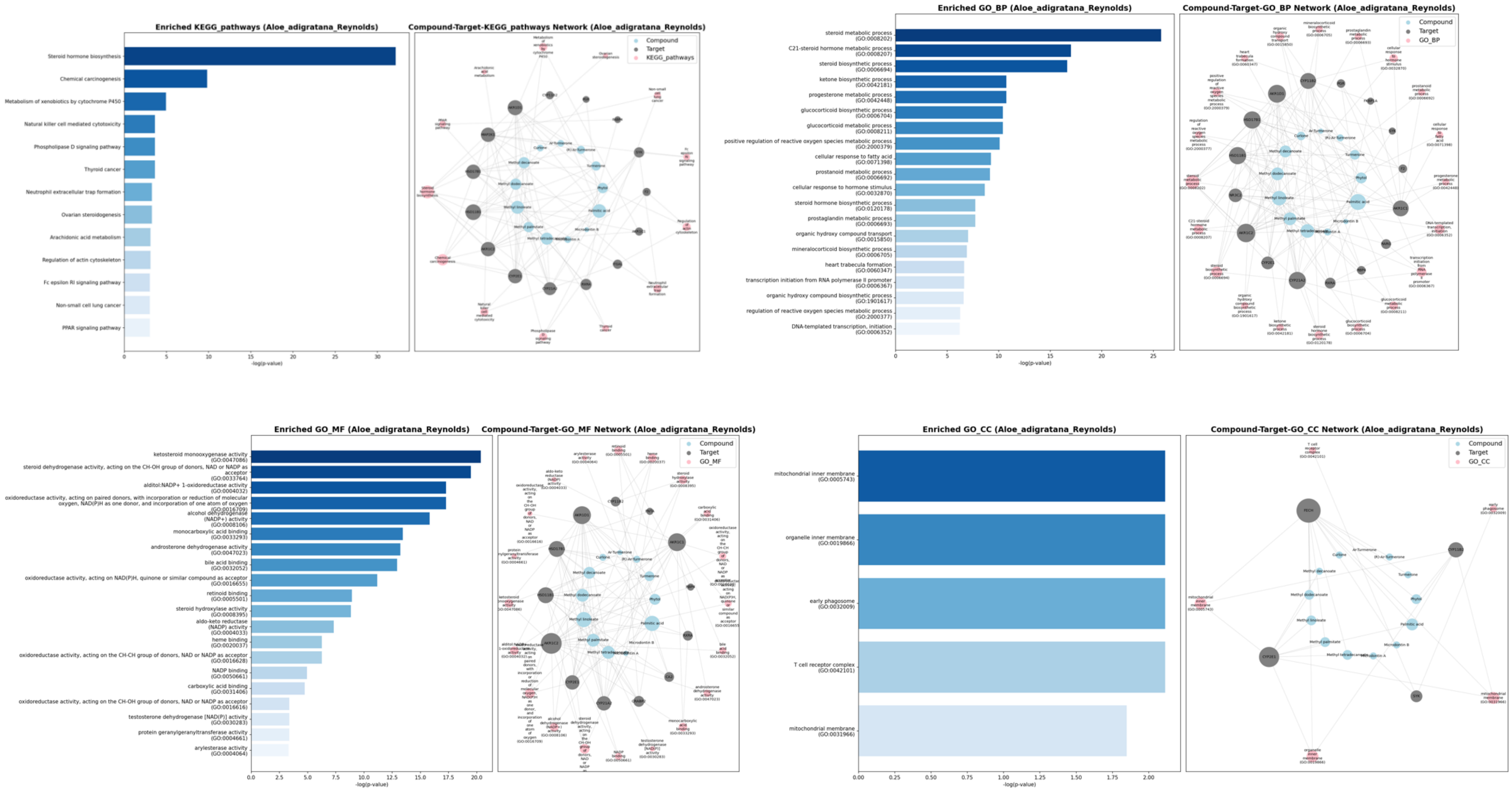

A. *camperi* Schweinf

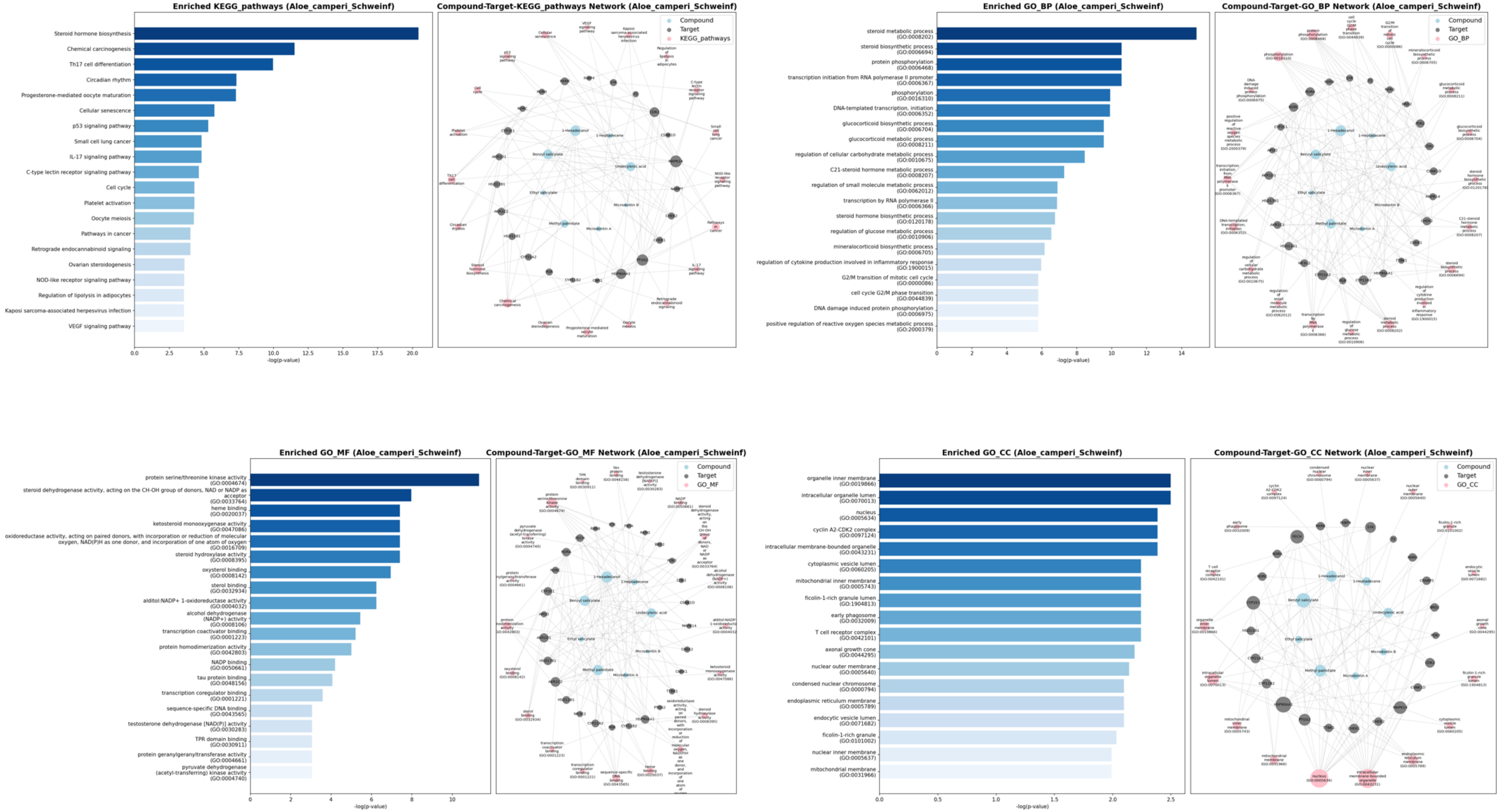

A. *debrana* Christian

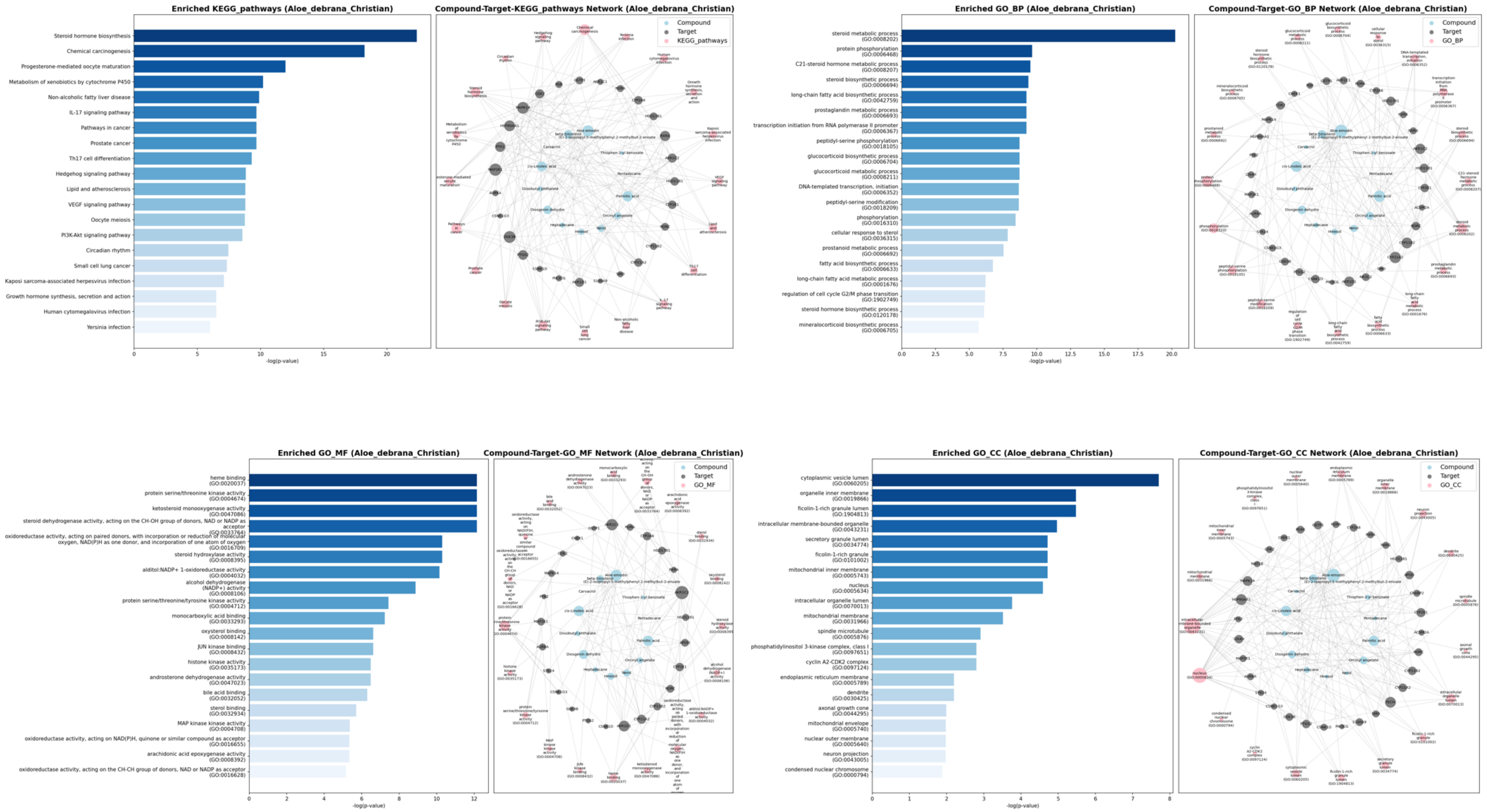

A. *elegans* Tod

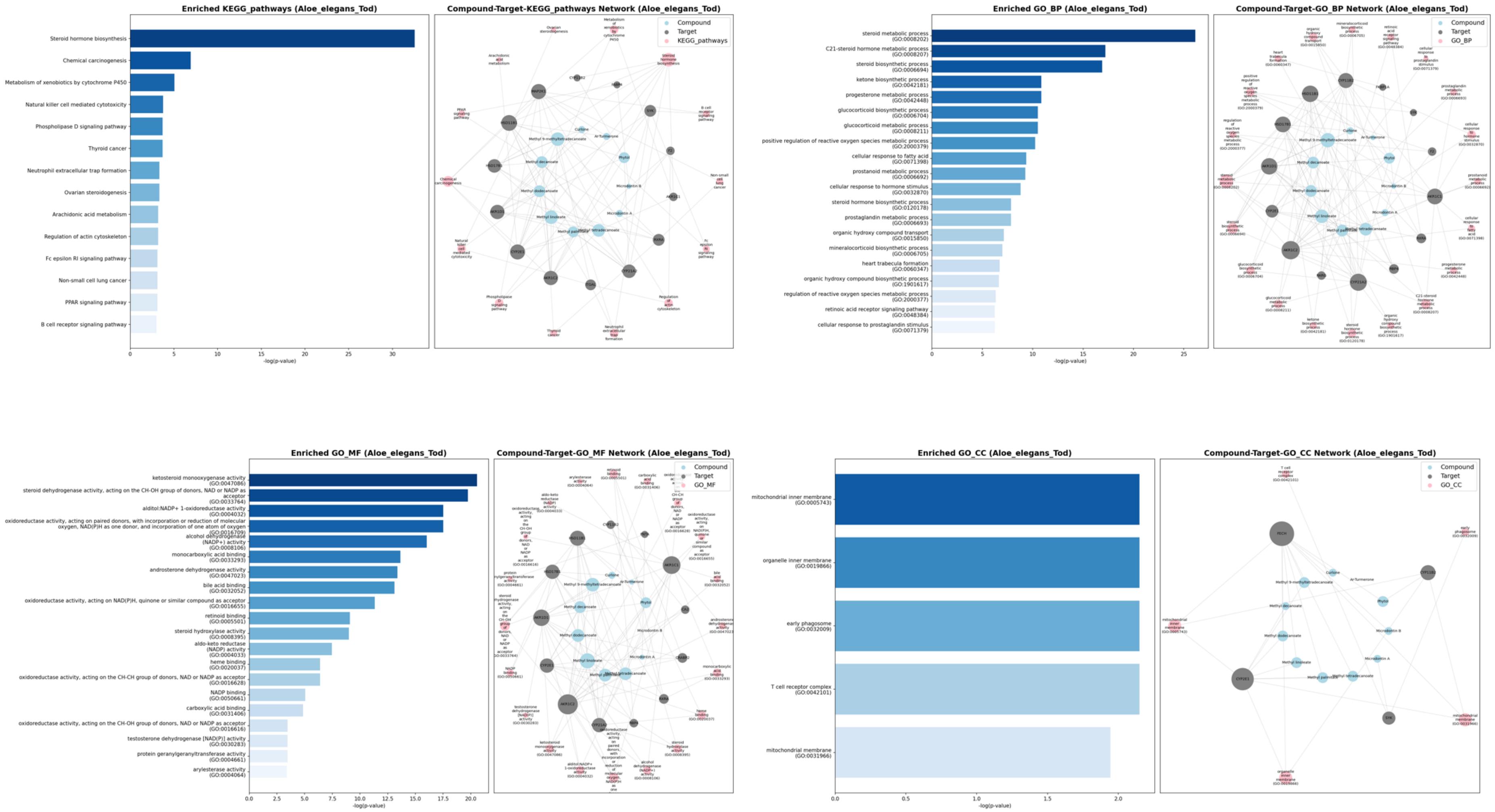

A. *gilbertii* Sebsebe and Brandham

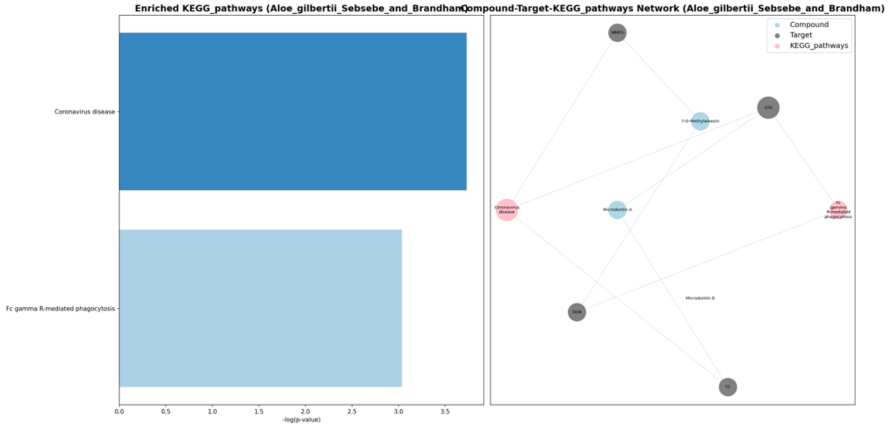

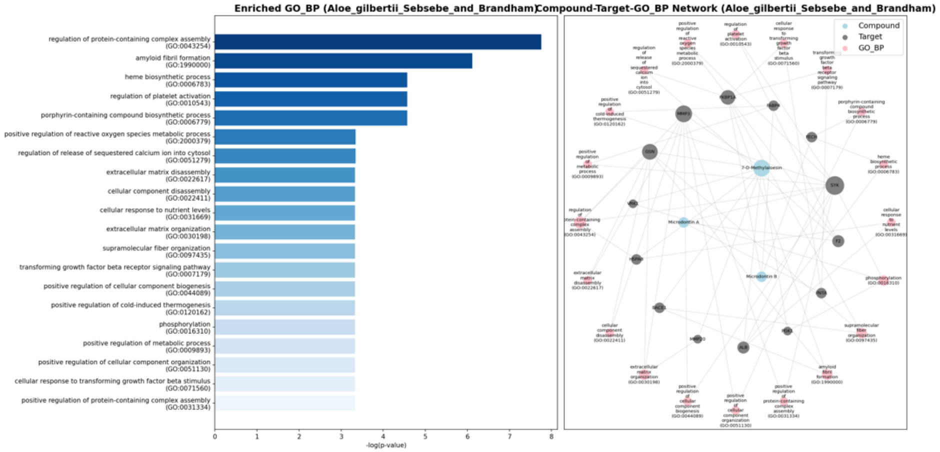

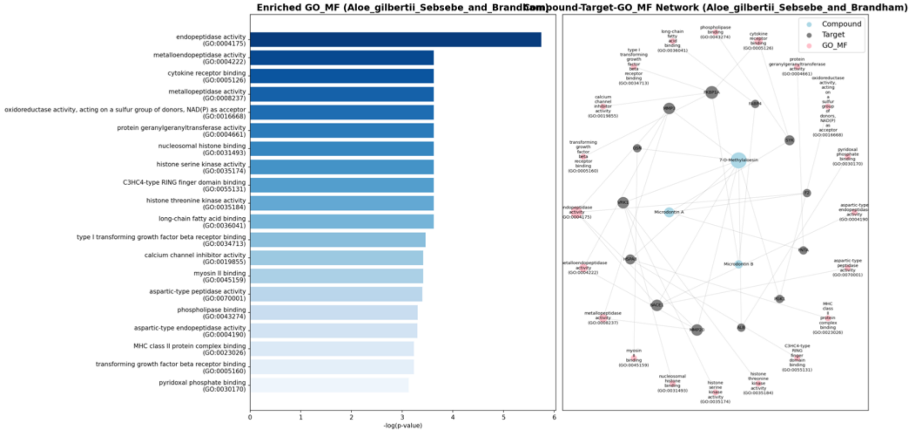

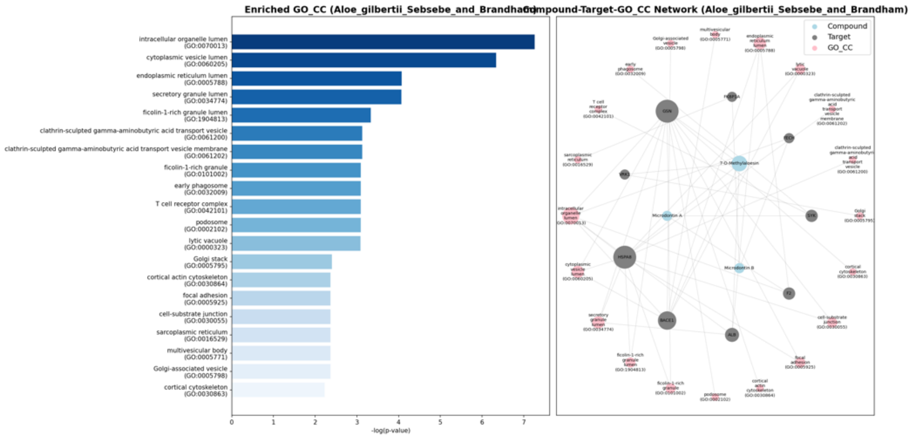

A. *harlana* Reynolds

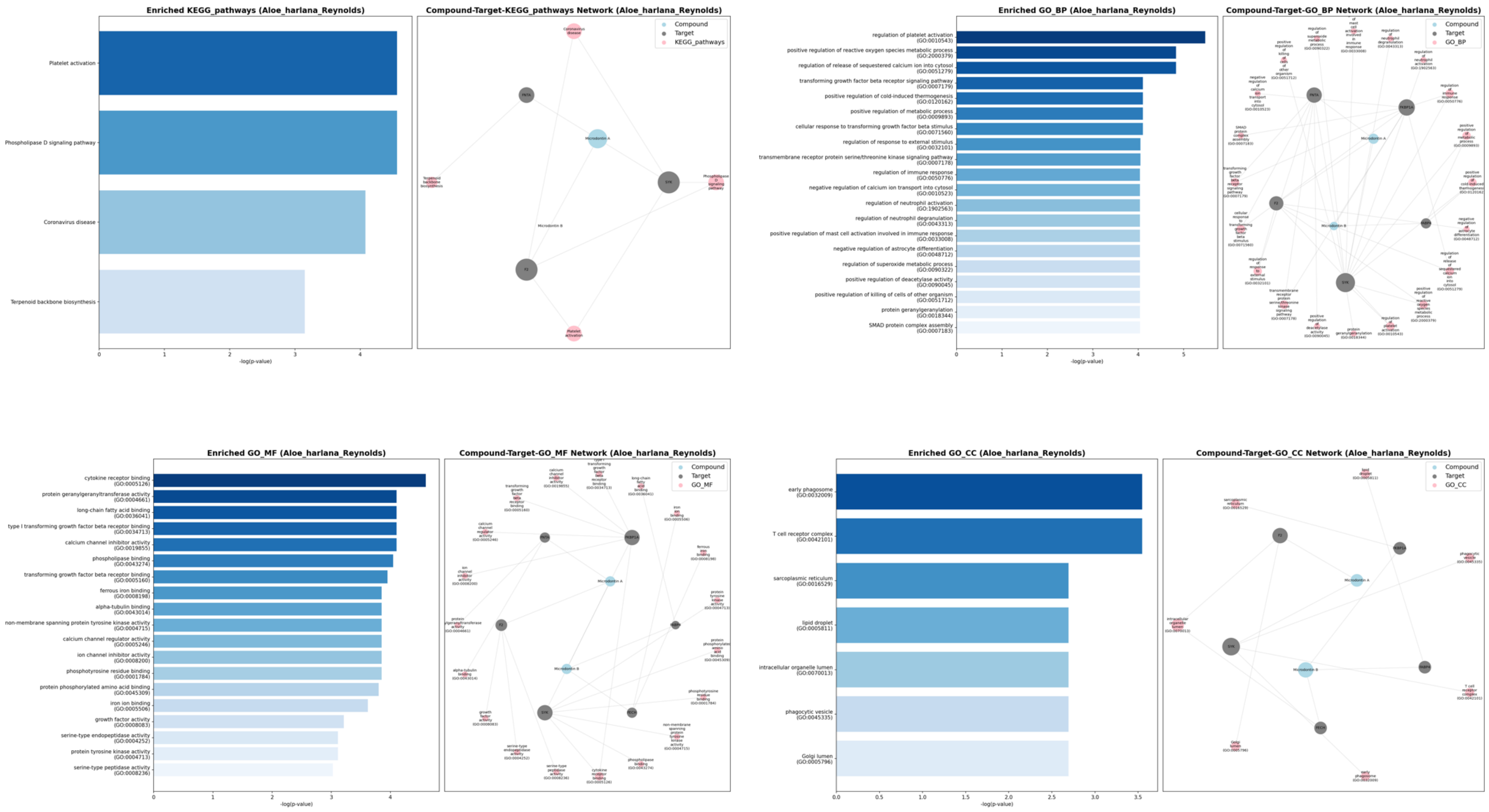

A. *jacksonii* Reynolds

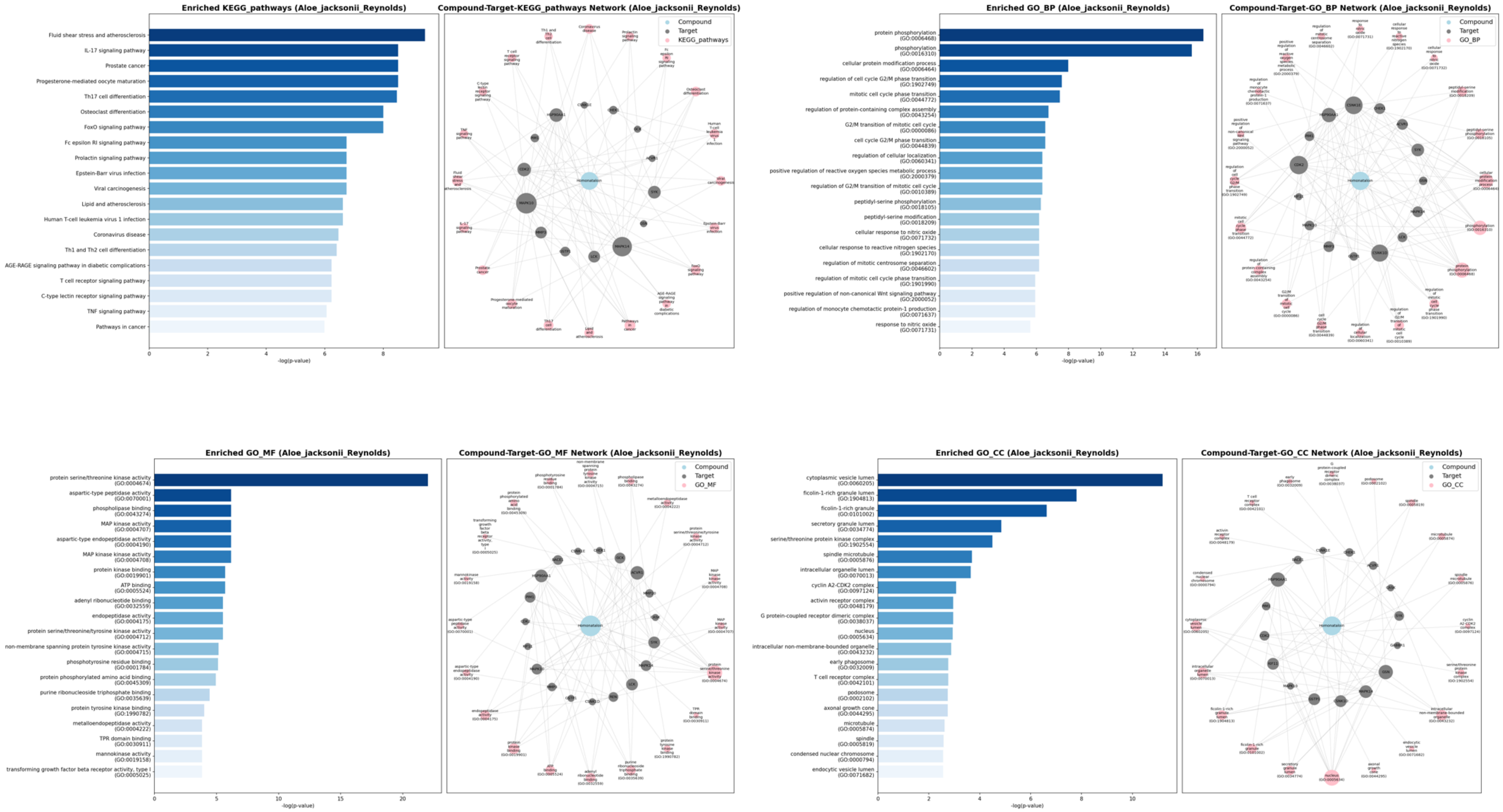

A. *megalacantha* Baker

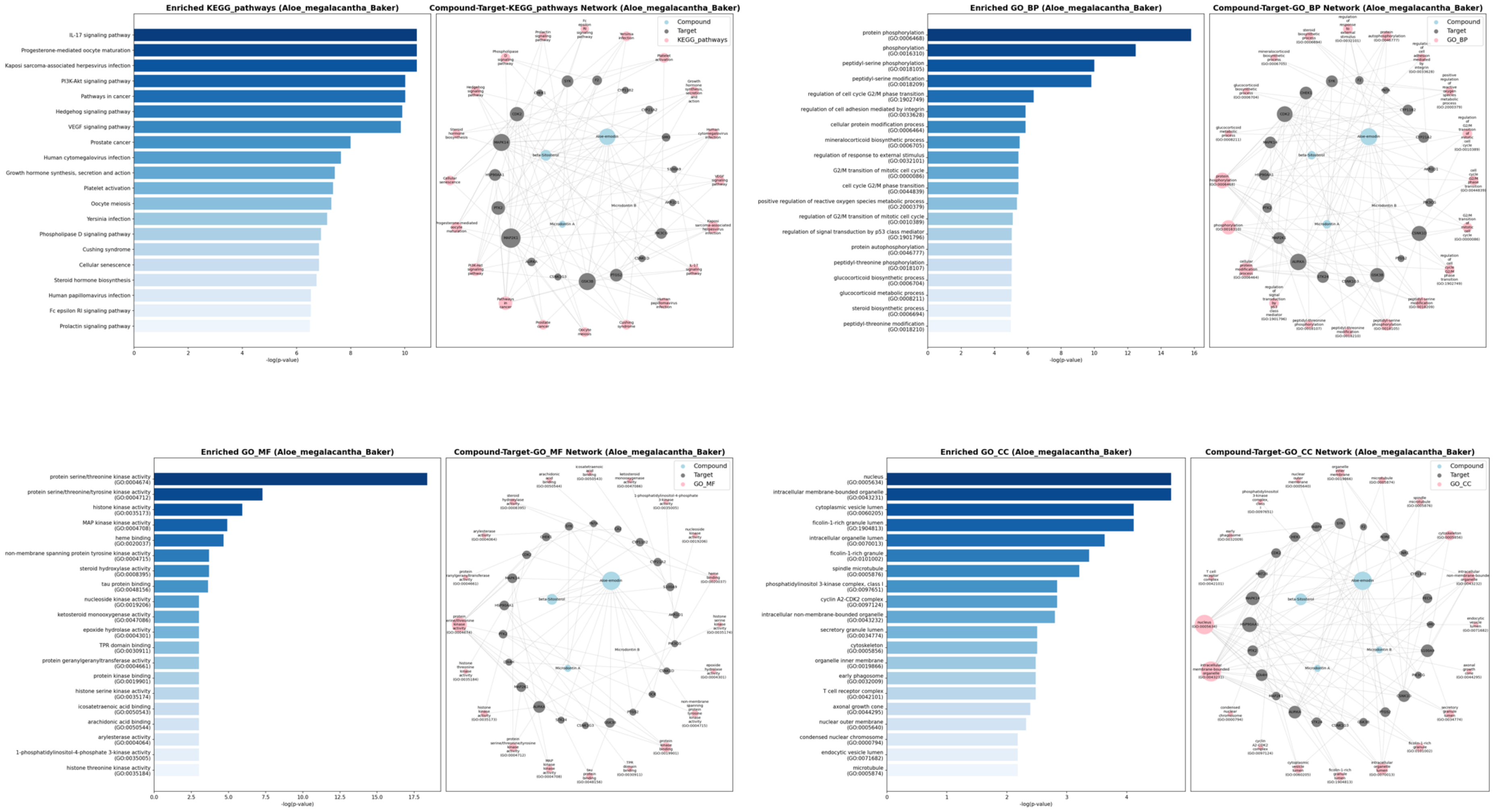

A. *monticola* Reynolds

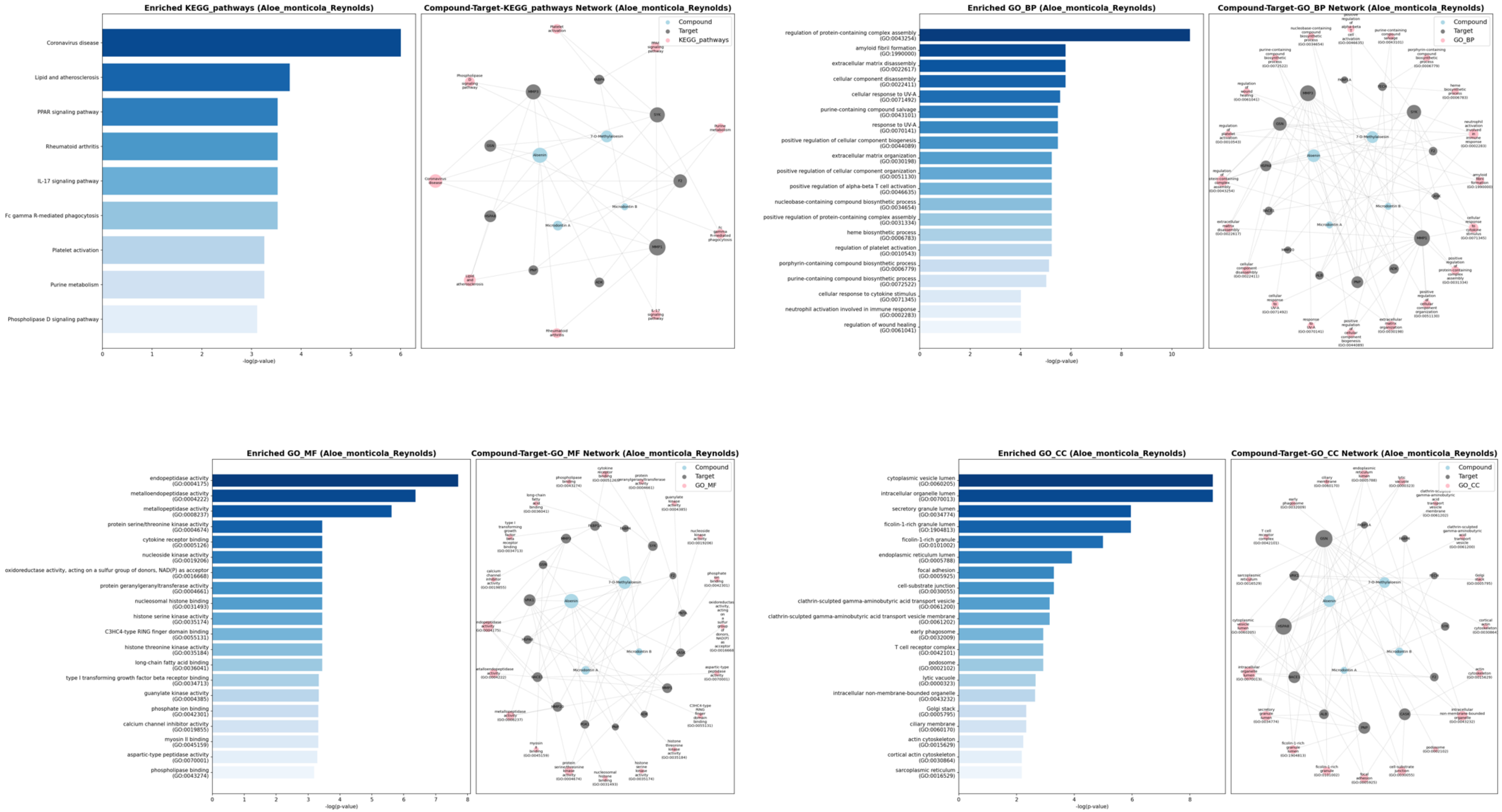

A. *schelpei* Reynolds

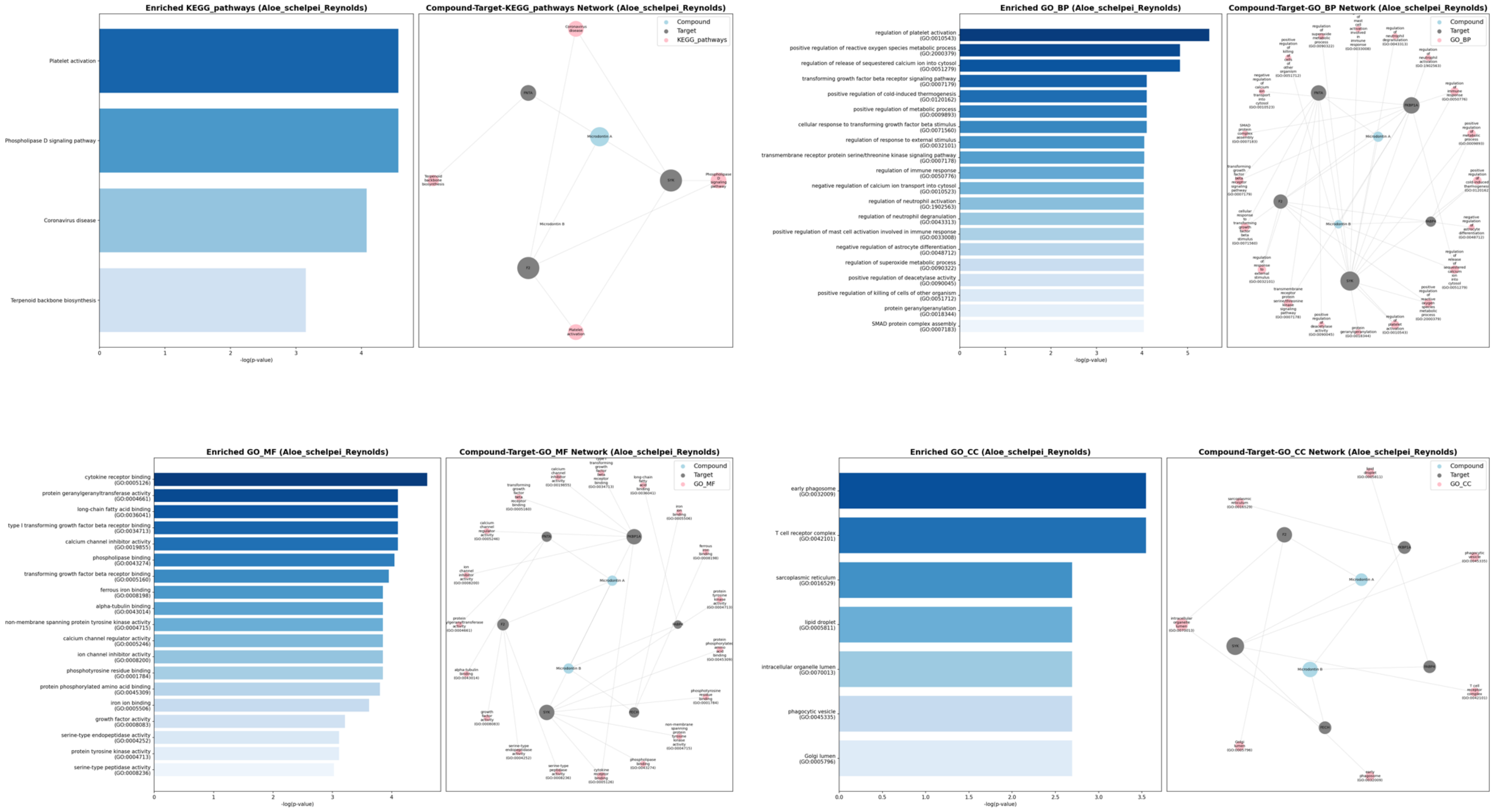

Supplement: Supplementary file 2 — Supplementary Information 2. [file 41598_2022_26446_MOESM2_ESM.pdf]
